# AAV-mediated gene augmentation therapy of *CRB1* patient-derived retinal organoids restores the histological and transcriptional retinal phenotype

**DOI:** 10.1016/j.stemcr.2023.03.014

**Published:** 2023-04-20

**Authors:** Nanda Boon, Xuefei Lu, Charlotte A. Andriessen, Ioannis Moustakas, Thilo M. Buck, Christian Freund, Christiaan H. Arendzen, Stefan Böhringer, Hailiang Mei, Jan Wijnholds

**Affiliations:** 1 Department of Ophthalmology, Leiden University Medical Center (LUMC), Albinusdreef 2, 2333 ZA Leiden, the Netherlands; 2 Sequencing Analysis Support Core, Department of Biomedical Data Sciences, Leiden University Medical Center (LUMC), Albinusdreef 2, 2333 ZA Leiden, the Netherlands; 3 hiPSC Hotel, Department of Anatomy and Embryology, Leiden University Medical Center (LUMC), Albinusdreef 2, 2333 ZA Leiden, the Netherlands; 4 Department of Biomedical Data Sciences, Leiden University Medical Center (LUMC), Albinusdreef 2, 2333 ZA Leiden, the Netherlands; 5 Netherlands Institute for Neuroscience, an Institute of the Royal Netherlands Academy of Arts and Sciences (KNAW), Meibergdreef 47, 1105 BA Amsterdam, the Netherlands

**Keywords:** CRB1, scRNA-seq, gene augmentation therapy, AAV.hCRB1, AAV.hCRB2, retinal organoids

## Abstract

Retinitis pigmentosa and Leber congenital amaurosis are inherited retinal dystrophies that can be caused by mutations in the Crumbs homolog 1 (*CRB1*) gene. CRB1 is required for organizing apical-basal polarity and adhesion between photoreceptors and Müller glial cells. *CRB1* patient-derived induced pluripotent stem cells were differentiated into *CRB1* retinal organoids that showed diminished expression of variant CRB1 protein observed by immunohistochemical analysis. Single-cell RNA sequencing revealed impact on, among others, the endosomal pathway and cell adhesion and migration in *CRB1* patient-derived retinal organoids compared with isogenic controls. Adeno-associated viral (AAV) vector-mediated h*CRB2* or h*CRB1* gene augmentation in Müller glial and photoreceptor cells partially restored the histological phenotype and transcriptomic profile of *CRB1* patient-derived retinal organoids. Altogether, we show proof-of-concept that AAV.h*CRB1* or AAV.h*CRB2* treatment improved the phenotype of *CRB1* patient-derived retinal organoids, providing essential information for future gene therapy approaches for patients with mutations in the *CRB1* gene.

## Highlights

• CRB1 variant protein, but not *CRB1* transcript, is diminished in *CRB1* organoids
• Gene ontology on *CRB1* organoids suggests an aberrant endosomal vesicular system
• AAV-mediated gene therapy partially restored the phenotype in *CRB1* organoids

## Teaser

Wijnholds and colleagues analyzed the phenotype and transcriptional differences between *CRB1* patient-derived organoids and isogenic controls. Gene profiling shows disruptions in the endosomal system in Müller glial cells and rods of *CRB1* patient-derived retinal organoids. They also showed a partially restored histological phenotype and transcriptional differences in the endosomal system after AAV-mediated *CRB* gene augmentation therapy.

## Introduction

Retinitis pigmentosa (RP) and Leber congenital amaurosis (LCA) are inherited retinal dystrophies caused by mutations in, among others, the Crumbs homolog 1 (*CRB1*) gene (Den Hollander et al., 1999; Nguyen et al., 2022; Talib et al., 2017). Canonical CRB1 is a large transmembrane protein consisting of a short 37 amino acid intracellular domain containing a protein 4.1, ezrin, radixin, moesin (FERM), and a conserved glutamic acid-arginine-leucine-isoleucine (ERLI) PDZ binding motif, a single transmembrane domain, a large extracellular domain with multiple epidermal growth factor and laminin-A globular-like domains (Boon et al., 2020; Den Hollander et al., 1999; Quinn et al., 2017). Recently, a short non-canonical alternatively spliced form of CRB1, *CRB1-B*, has been described containing substantial extracellular domain overlap but with distinct amino terminus and lacking the C-terminal transmembrane and intracellular domains (Ray et al., 2020). In mammals, canonical CRB1 is a member of the Crumbs family together with CRB2 and CRB3A. The canonical CRB complex is formed by interaction with protein associated with Lin seven 1 (PALS1), also known as membrane-associated guanylate kinase p55 subfamily member 5 (MPP5), which binds to the conserved C-terminal PDZ domain of CRB (Margolis, 2018; van de Pavert et al., 2004; Roh et al., 2002). Binding of PALS1 can lead to the recruitment of multiple PDZ domain protein 1 (MUPP1) or the InaD-like protein (INADL/PATJ) to the apical membrane (Roh et al., 2002). This CRB complex is evolutionary conserved and is important for maintaining cell adhesion and regulating apical-basal polarity (Bulgakova and Knust, 2009).

So far, no treatment possibilities are available for patients with RP or LCA caused by mutations in *CRB1.* Gene augmentation therapies using adeno-associated viral (AAV) vectors are of emerging interest for retinal dystrophies because of the recent FDA approval of an AAV-mediated gene therapy approach for RP and LCA patients with mutations in the *RPE65* gene (Maguire et al., 2019). AAVs are the leading platform for gene delivery because of their low toxicity, limited integration into the host genome, and because different AAV capsids display distinct cell tropisms. Their major disadvantage is the limited packaging capacity; inverted terminal repeats, cDNAs, and regulatory sequences bigger than 4.9 kb often do not fit in a single AAV capsid. Unfortunately, the full-length cytomegalovirus (CMV) ubiquitous promotor and h*CRB1* cDNA exceeds this packaging limit. However, substantial expression levels of canonical hCRB1 protein in mouse mutant *Crb1* retina were observed using an AAV with codon optimized h*CRB1* cDNA linked to a minimal CMV promoter (Pellissier et al., 2014a). This AAV.CMVmin.h*CRB1* was deleterious upon intravitreal injection in *Crb1* mouse models (Pellissier et al., 2015). As an alternative approach, *CRB* family member *CRB2* was used to restore retinal function and vision in *Crb* mice (Buck et al., 2021; Pellissier et al., 2015), showing the potential of AAV.h*CRB2* gene augmentation therapy for patients with mutations in the *CRB1* gene.

There are several mouse models described with mutations in the *Crb1* and/or *Crb2* gene mimicking the RP or LCA phenotype (Alves et al., 2013, Alves et al., 2014, Alves et al., 2019; Mehalow et al., 2003; van de Pavert et al., 2004, 2007a; Pellissier et al., 2013; Quinn et al., 2018, 2019a). However, immunoelectron microscopy identified the subcellular localization of CRB1 and CRB2 proteins to be different in mouse and human models. In mice, CRB2 is present in photoreceptor cells and Müller glial cells (MGCs) at the subapical region (SAR) of the outer limiting membrane (OLM), while CRB1 is solely present in MGCs at the SAR (van Rossum et al., 2006). In contrast, in human fetal retina and human induced pluripotent stem cell (hiPSC)-derived retinal organoids both CRB1 and CRB2 are observed at the SAR in photoreceptors and MGCs (Quinn et al., 2019b). This discrepancy suggests the importance of using human-derived models for gene therapy approaches.

Here, we describe the phenotype of differentiation day 210 (DD210) and DD230 patient-derived retinal organoids harboring *CRB1* missense mutations compared with isogenic controls in more detail using immunohistochemical analysis and single-cell RNA sequencing (scRNA-seq). Next, the effect of AAV-mediated h*CRB1* or h*CRB2* gene augmentation therapy was analyzed on *CRB1* patient-derived and isogenic control retinal organoids. A partially improved retinal phenotype of *CRB1* patient-derived retinal organoids was observed, providing crucial data for future gene therapy approaches for patients with mutations in the *CRB1* gene.

## Results

### Reduced number of photoreceptor nuclei and thinner outer nuclear layer in DD210 *CRB1* patient-derived retinal organoids compared with isogenic controls

Retinal organoids were differentiated from hiPSC lines derived from three *CRB1* RP patients: (1) LUMC0116iCRB with c.3122T>C p.(Met1041Thr) homozygote missense mutations (here abbreviated as P116), (2) LUMC0117iCRB with 2983G>T p.(Glu995∗) and c.1892A>G, p.(Tyr631Cys) mutations (P117), and (3) LUMC0128iCRB with c.2843G>A p.(Cys948Tyr) and c.3122T>C p.(Met1041Thr) missense mutations (P128) (Quinn et al., 2019b). Isogenic controls for P116 and P128 were generated by CRISPR-Cas9; (1) ISO-02 P116 with a homozygous correction, (2) ISO-03 P116 with a heterozygous correction, and (3) ISO-P128 a heterozygous correction of Cys948Tyr (Table S1). Genomic stability of all iPSC lines was tested by a digital PCR test of the copy number variants (CNVs) of 90% of the most recurrent abnormalities in hiPSCs (Assou et al., 2020). No aberrant CNVs were observed in the hiPSC lines used in this study (Figure S1A).

**Figure 1:**
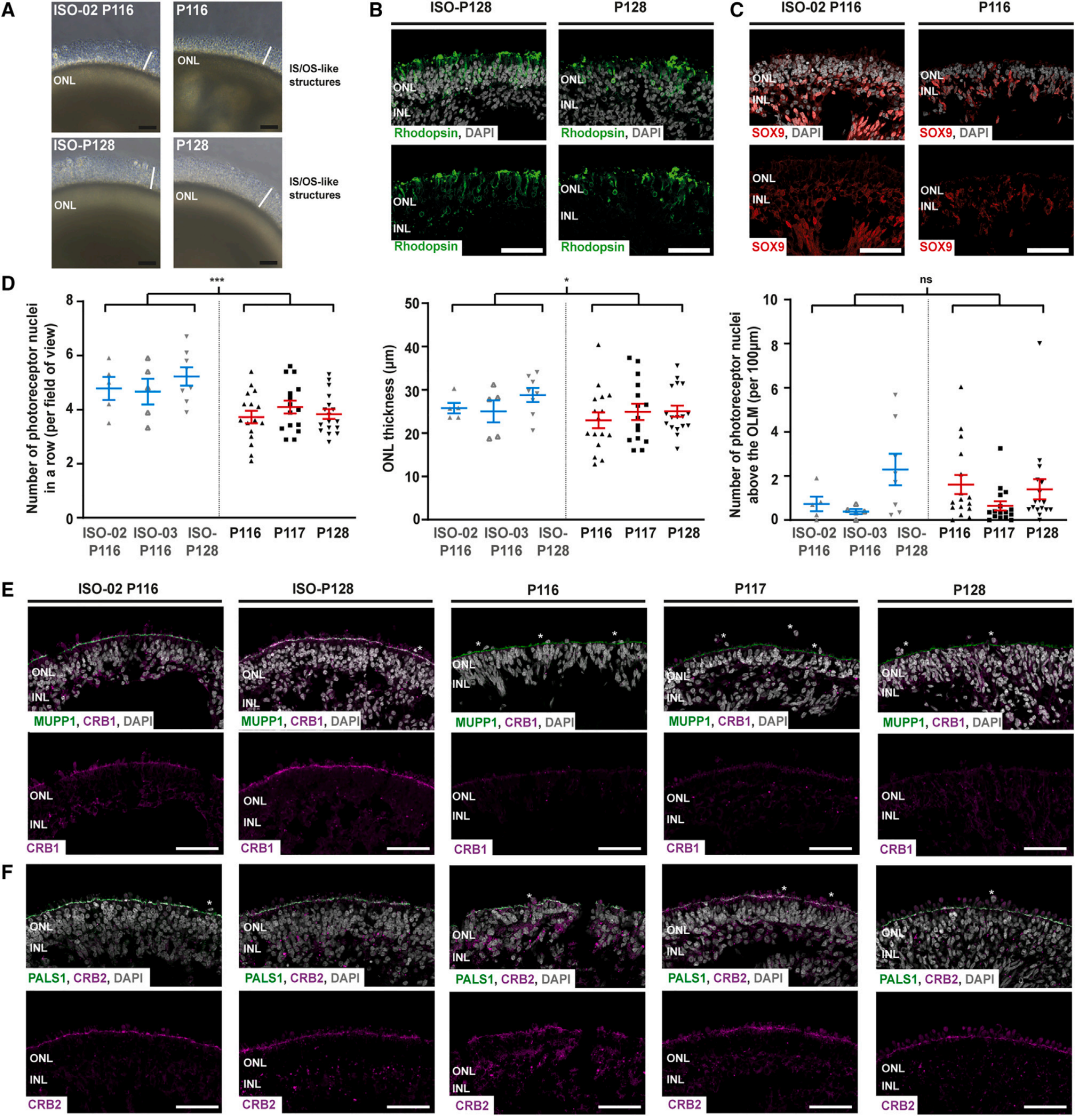
*CRB1* patient and isogenic control phenotypic analysis at DD210 (A) Representative bright-field images of ISO-02 P116, P116, ISO-P128, and P128 cultured organoids. (B) Representative immunohistochemical images of rhodopsin (green) in ISO-P128 and P128. (C) Representative immunohistochemical images of SOX9 (red) in ISO-P116 and P116. (D) Quantitative analysis of number of photoreceptor nuclei in a row per field of view (p = 0.000), ONL thickness per field of view (p = 0.049), and number of photoreceptor nuclei above the OLM per 100 μm (p = 0.651) in *CRB1* patient-derived and isogenic control retinal organoids. (E) Immunohistochemical images of CRB1 (red) and MUPP1 (green) in *CRB1* patient-derived retinal organoids with two appropriate isogenic controls. (F) Immunohistochemical images of CRB2 (red) and PALS1 (green) in *CRB1* patient-derived retinal organoids with two appropriate isogenic controls. Each datapoint in the graph represents individual organoids, of which an average has been taken of 3–6 representative images. The standard error of mean (SEM) is derived from these averages. Number of individual organoids per condition and differentiation round: P116, n = 16; P117, n = 15; P128, n = 17, from four independent organoid batches; ISO-P128, n = 8 from three independent organoid batches; ISO-02 P116, n = 5; and ISO-03 P116, n = 5 from two independent organoid batches. Scale bars, 50 μm. Statistical analysis: generalized linear mixed models with ^∗^p < 0.05, ^∗∗^p < 0.01, ^∗∗∗^p < 0.001. Related to Figure S1.

Previous research has shown that *CRB1* patient-derived retinal organoids at DD180 show disruptions at the OLM and photoreceptor nuclei protruding above the OLM (Quinn et al., 2019b). Here, we analyzed the phenotype of the *CRB1* patient-derived retinal organoids at a later timepoint (DD210) and compared those with the isogenic controls. By light microscopy, no visible difference was observed in cultured retinal organoids comparing *CRB1* patient with the isogenic controls at DD210: all contained a translucent region at the outside of the organoid (the ONL) with inner and/or outer-segment-like structures around the retinal organoid (Figures 1A and S1B). Immunohistochemistry of rod photoreceptor marker rhodopsin and MGC marker SOX9 at DD210 revealed the presence of rod photoreceptors and SOX9-positive MGCs for both patient and isogenic controls (Figures 1B, 1C, S1C, and S1D).

When analyzing the phenotype in more detail, a moderate but statistically significant decrease in the number of photoreceptor nuclei and ONL thickness was observed in *CRB1* patient-derived retinal organoids compared with isogenic controls (Figure 1D). In contrast to *CRB1* patient-derived retinal organoids at DD180 (Quinn et al., 2019b), no statistically significant difference in the number of photoreceptor nuclei above the OLM was detected at DD210 (Figure 1D). In addition, no statistically significant differences were observed for the total retinal thickness and the inner nuclear layer (INL) thickness of the retinal organoids (Figure S1H).

### Missense mutations in *CRB1* result in reduced levels of variant CRB1 protein in *CRB1* patient-derived retinal organoids

Immunohistochemistry analysis of all three *CRB1* patient-derived retinal organoids at DD210 shows a diminished CRB1 staining at the OLM compared with the isogenic control (Figures 1E and S1E). Similar strongly diminished levels of variant CRB1 in *CRB1* patient-derived retinal organoids compared with the isogenic control were observed at DD180 (Figure S1G), while CRB2 and CRB complex members MUPP1 and PALS1 remain at the OLM in both isogenic and *CRB1* patient-derived organoids (Figures 1E, 1F, and S1F).

### Missense mutations in CRB1 do not affect the levels of CRB1 or CRB2 RNA transcripts in *CRB1* patient-derived retinal organoids

**Figure 2:**
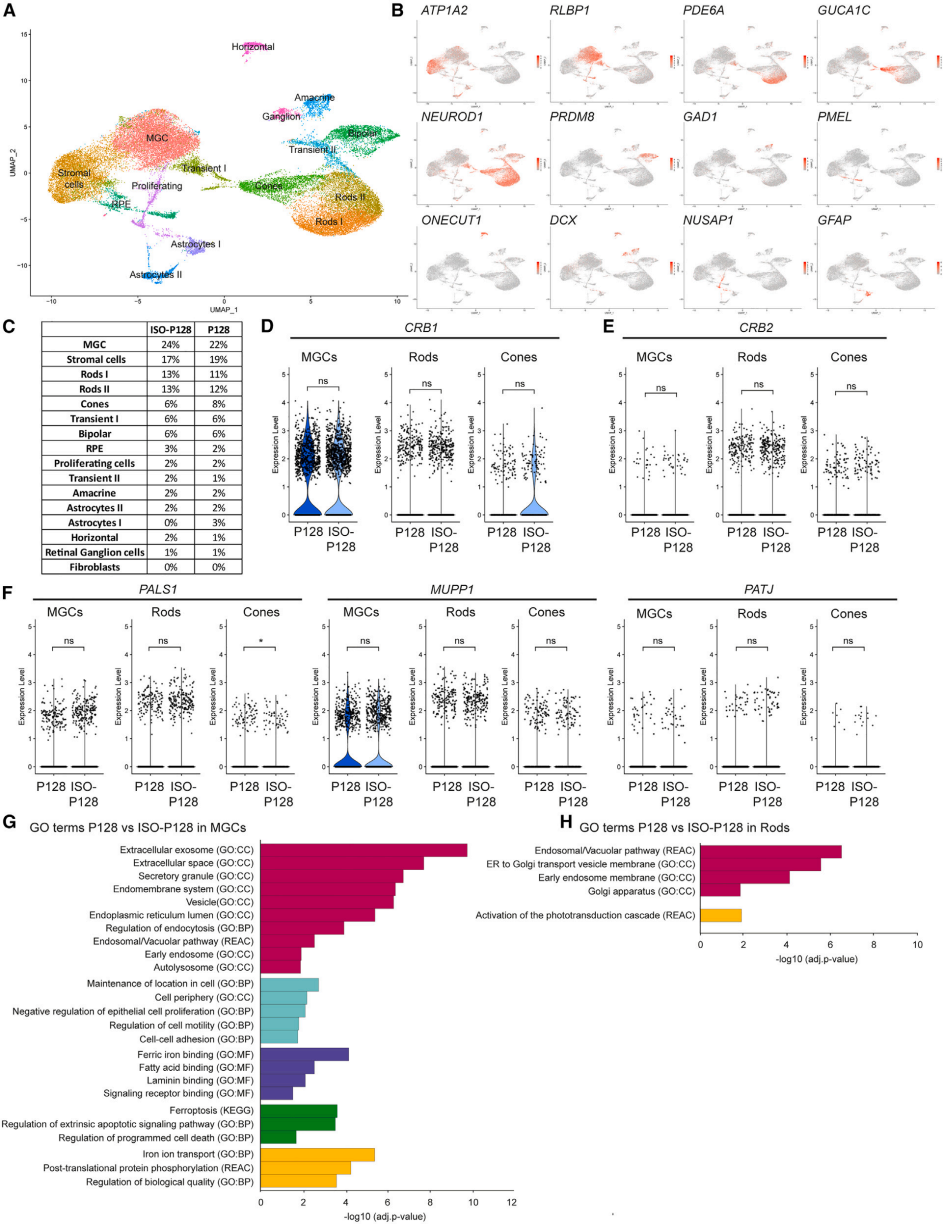
scRNA-seq analysis comparing ISO-P128 with P128 shows disruptions in the endosomal system in Müller glial cells and rods (A and B) (A) UMAP plot of observed clusters and (B) expression plots of top markers indicating the distinct clusters. (C) Table showing that all retinal cell types are present in both lines. (D) Violin plots of *CRB1* transcript levels specifically in MGCs (p = 0.93), rods (p = 0.84), and cones (p = 0.72). (E) Violin plots of *CRB2* transcript levels specifically in MGCs (p = 0.43), rods (p = 0.18), and cones (p = 0.85). (F) Violin plots of canonical core CRB complex members *PALS1*, *MUPP1*, and *PATJ* transcript levels in MGCs (p = 0.32; p = 0.51; p = 0.73, respectively), rods (p = 0.97; p = 0.18; p = 0.63), and cones (p = 0.025; p = 0.25; p = 0.87). (G and H) Gene ontology (GO) analysis of differentially expressed markers specifically in MGCs (G) and rods (H) clustered in groups with similar terms in the same color. Number of independent organoids used: ISO-P128, n = 6; and P128, n = 6, from one differentiation round equally divided into three separate sequencing rounds. Related to Figures S2 and S3 and Table S4.

Next, we used scRNA-seq to identify differences in RNA transcripts and Gene Ontology (GO) pathways between DD230 *CRB1* patient-derived retinal organoids and isogenic controls. Transcriptionally similar cells were grouped and visualized (R package Seurat), revealing distinct clusters with differentially expressed marker genes (Table S3). These identified expressed genes per cluster were compared with known retinal marker genes to classify clusters. Major retinal cell types could be visualized on a UMAP plot, such as MGCs, photoreceptor cells (both rods and cones), bipolar cells, amacrine cells, horizontal cells, ganglion cells, and retinal pigment epithelium (RPE) (Figure 2A). In addition, some of the clusters consisted of astrocytes and transitory cells (Figure 2A), and tissue that is generally attached to the retinal organoid was observed and classified as stromal cells (Figures 2A and S2C). The expression of key cell-type-specific markers of all clusters are shown in a feature plot and heatmap (Figures 2B and S2A). Interestingly, two rod photoreceptor cell subtypes can be distinguished. Upon further analysis, cluster rods I was identified to be composed of more mature cells with significantly higher transcript expression levels of *NR2E3*, *PDE6B*, and *RHO* in comparison with cluster rods II (Figure S2B) (Swaroop et al., 2010).

We confirmed that all the major retinal cell clusters were equally present in both P128 and ISO-P128 (Figure 2C) and P116 and ISO-P116 (Figure S3A). Expression levels of both *CRB1* and *CRB2* were predominantly observed in MGCs and photoreceptor cells. When analyzing *CRB1* expression levels in MGCs and photoreceptor cells in more detail, no statistically significant differences were observed between P128 and ISO-P128 (Figure 2D) or between P116 and ISO-P116 (Figure S3B). Rods I and rods II were combined into a general “rods” cluster, since no statistically significant differences were observed in the individual clusters (data not shown). Moreover, the sequence of *CRB1-B*, an alternative transcript of *CRB1* containing distinct 5′ and 3′ ends, was added to the pre-built reference and was not detected in our DD230 retinal organoids (Figure S2D). While *CRB1-B* transcripts were detected in adult human retina cDNA, the levels of *CRB1-B* transcripts were below detection level by quantitative reverse transcription real-time PCR (qRT-PCR) on DD210 retinal organoids (data not shown; experimental procedures in supplemental information). In addition, no statistically significant difference was observed for the *CRB2* expression level (Figures 2E and S3C), canonical CRB core complex members *PALS1*, *MUPP1*, *PATJ* (Figures 2F and S3D), or FERM proteins *MSN*, *EZR*, or *EPB41L5* (Figure S2E).

### Gene profiling shows disruptions in the endosomal system in MGCs and rods

Differential gene expression analysis followed by GO term analysis comparing *CRB1* patient and isogenic control retinal organoids was performed. Analysis of P128 and ISO-P128 specifically in the MGCs, where most *CRB1* is expressed, revealed five groups of similar GO terms containing differentially expressed genes deregulated in the patient-derived retinal organoids. The first GO group is involved in the endosomal system, including extracellular exosomes, vesicles, endomembrane system, and early endosomes (Figure 2G). The second group is involved in the maintenance of location in the cell, cell motility, proliferation, and cell-cell adhesion, the third group revealed differences in proteins containing various binding domains such as ferric iron and fatty acid, while the fourth group is involved in cell death (Figure 2G). Finally, the last one is a mixed group with pathways such as iron ion transport and post-translational protein phosphorylation (Figure 2G). In addition, as the *CRB1* transcript is also present in photoreceptor cells, differentially expressed markers and subsequent GO terms were analyzed in rods (combination of rods I and rods II) and cones. In rods, GO terms involved in the endosomal system were observed to be differentially expressed (Figure 2H). In addition, markers associated with the activation of the phototransduction cascade were detected (Figure 2H). No statistically significant differential expressed markers were observed in cone photoreceptor cells (data not shown).

Such an analysis was also performed comparing P116 with ISO-P116, where GO terms associated with the endosomal system were differentially expressed in rod photoreceptor cells (Figure S3E). No statistically significant GO terms were observed in MGCs, explained by the low number of differentially expressed genes and the low number of cells sequenced in this cluster. Altogether, these data show aberrations in the endosomal system between *CRB1* patient-derived retinal organoids compared with their isogenic controls.

### Serotype AAV5.CMV.*GFP* is more efficient than AAV2.CMV.*GFP* in transducing MGCs at DD120

CRB1 protein is localized at the OLM in human and non-human primate MGCs and photoreceptors (Quinn et al., 2019a, 2019b), and higher levels of *CRB1* transcript are found in MGCs than in photoreceptors (Figure 2D). For AAV-mediated gene therapy approaches in *CRB1* patient-derived retinal organoids it is therefore essential to transduce a sufficient number of MGCs in addition to photoreceptors. Therefore, we identified which cells are transduced using specific viral capsids and titers at DD120. Control retinal organoids were transduced with 1 × 10^10^ genome copies (gc), 6,6 × 10^10^ gc, or 10 × 10^10^ gc AAV2/5.CMV.*GFP* (AAV5.CMV.*GFP*) or AAV2/2.CMV.*GFP* (AAV2.CMV.*GFP*) and analyzed using immunohistochemistry after 3 weeks in culture.

**Figure 3:**
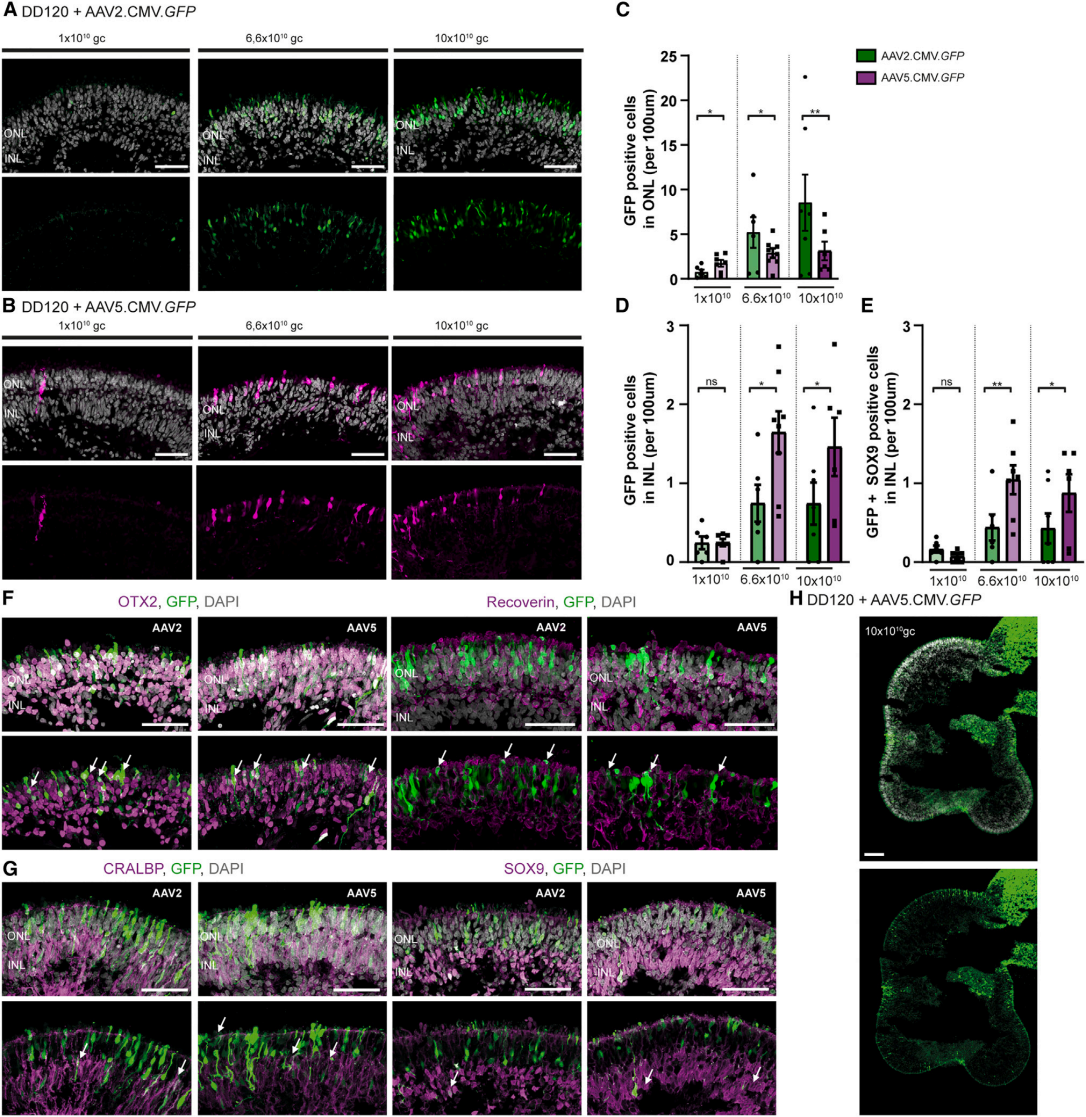
AAV2.CMV.*GFP* and AAV5.CMV.*GFP* transduction of DD120 control retinal organoids (A and B) Representative immunohistochemical images of (A) AAV2.CMV.*GFP-* and (B) AAV5.CMV.*GFP*-treated control organoids at DD120 with three different titer concentrations: 1 × 10^10^, 6.6 × 10^10^, and 10 × 10^10^ genome copies (gc). (C–E) Quantification of AAV-treated retinal organoids with AAV2.CMV.*GFP* or AAV5.CMV.*GFP* at the (C) ONL, (D) INL, or (E) GFP-positive MGC in the INL. (F and G) Representative immunohistochemical images of photoreceptor cell markers (OTX2 and recoverin) and MGC markers (CRALBP and SOX9) showing colocalization with AAV.*GFP* for both AAV2.CMV.*GFP-* and AAV5.CMV.*GFP*-treated organoids. (H) Representative 10× magnification immunohistochemical analysis of DD120 control organoid transduced with 10 × 10^10^ gc AAV5.CMV.*GFP*. Immunohistochemical images of (F, G, and H) are merged z stack views, the others are single image views. Scale bars, 50 μm. Each datapoint in the graph represents individual organoids, of which an average has been taken of at least three representative images. The standard error of mean (SEM) is derived from these averages. Number of individual organoids per condition: for AAV2.CMV.*GFP* 1 × 10^10^ n = 5, 6.6 × 10^10^ n = 6, and 10 × 10^10^ n = 7, and for AAV5.CMV.*GFP* 1 × 10^10^ n = 7, 6.6 × 10^10^ n = 8, and 10 × 10^10^ n = 6 individual organoids from two independent differentiation rounds. Statistical analysis: generalized linear mixed models with ^∗^p < 0.05, ^∗∗^p < 0.01, ^∗∗∗^p < 0.001. Related to Figure S3.

A significant dose-dependent increase of GFP-positive cells was observed when control organoids were treated with AAV5.CMV.*GFP* or AAV2.CMV.*GFP* at DD120 (Figures S3F and S3G). The AAV-treated retinal organoids were quantified for number of GFP-positive cells in the ONL, the INL, and GFP-positive cells in the INL that were also SOX9 positive (marking MGCs). AAV2.CMV.*GFP* transduced more photoreceptor cells in the ONL than AAV5.CMV.*GFP* (Figure 3C). However, AAV5.CMV.*GFP* transduced more cells in the INL than AAV2.CMV.*GFP* (Figure 3D). More specifically, more SOX9-positive MGCs were transduced with AAV5.CMV.*GFP* than with AAV2.CMV.*GFP* (Figure 3E). Co-staining with photoreceptor markers (OTX2 and recoverin) and MGC markers (CRALBP and SOX9) confirmed the transduction of both cell types in AAV2.CMV.*GFP* as well as AAV5.CMV.*GFP* transduced organoids at DD120 (Figures 3F and 3G). Moreover, a 10× magnification of a retinal organoid treated with 10 × 10^10^ gc AAV5.CMV.*GFP* at DD120 showed that most of the retinal organoid was transduced in our experiment (Figure 3H).

Because AAV5 transduced more MGCs than AAV2, treatment with AAV5 at DD120 was used for further AAV.h*CRB* gene augmentation therapy with an intermediate dose of 3.3 × 10^10^ gc.

### AAV-mediated h*CRB* gene augmentation therapy partially restores the histological phenotype of *CRB1* patient-derived retinal organoids

After defining the AAV.*GFP* tropism, preclinical gene therapy approaches were performed on *CRB1* patient-derived retinal organoids using AAV5.CMVmin.h*CRB1* or AAV5.CMV.h*CRB2* (here abbreviated as AAV.h*CRB1* and AAV.h*CRB2*, respectively) at DD120 and analyzed at DD180 or DD210.

Immunohistochemical analysis and subsequent quantification of retinal organoids transduced with AAV.h*CRB1* or AAV.h*CRB2* and analyzed at DD180 show an increased number of photoreceptor nuclei in a row in AAV.*CRB*-treated P117 compared with the control (Figure S4B). In addition, fewer photoreceptor nuclei protruding above the OLM were observed after AAV.*CRB* treatment of P117 compared with the control organoids (Figure S4C). No statistically significant difference was observed for the retinal or the ONL thickness in both *CRB1* patient and control organoids treated with AAV.h*CRB1* or AAV.h*CRB2* (Figures S4D and S4E).

**Figure 4:**
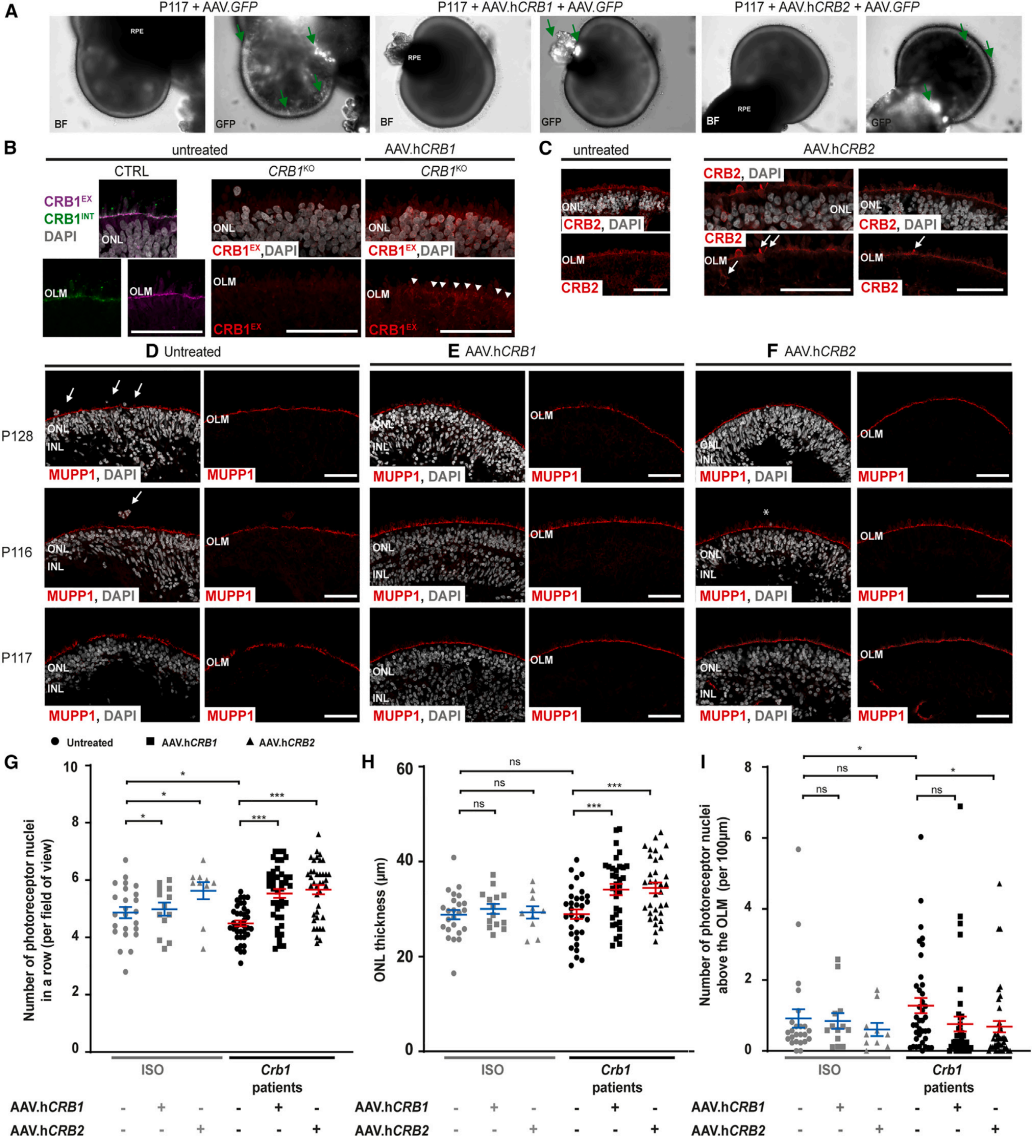
AAV-mediated gene therapy treatment on *CRB1* patient-derived and isogenic control retinal organoids (A) Representative bright-field (BF) and fluorescent (GFP regions indicated with green arrow) images of DD210 cultured P117 retinal organoids treated with 3.3 × 10^10^ vg AAV.h*CRB*. (B) Immunohistochemical image of CRB1 in an untreated control retinal organoid, an untreated *CRB1*^KO^ retinal organoid, and an AAV.h*CRB1*-treated *CRB1*^KO^ retinal organoid showing increased CRB1 localization at the OLM of AAV.h*CRB1*-treated *CRB1*^KO^ retinal organoids (arrowheads). (C) Immunohistochemical image of CRB2 in untreated and AAV.h*CRB2*-treated *CRB1* patient-derived retinal organoid at the OLM. Arrows indicate overexpression of CRB2 in photoreceptor cells in AAV.h*CRB2*-treated retinal organoids. (D–F) Representative immunohistochemical images of (D) untreated, (E) AAV.h*CRB1*-, and (F) AAV.h*CRB2*-treated *CRB1* patient retinal organoids stained with MUPP1 (red) at DD210. (G) Quantification of the number of photoreceptor nuclei in a row (from left to right: p = 0.000, p = 0.000, p = 0.039, p = 0.046, p = 0.046), (H) ONL thickness (p = 0.001, p = 0.001, p = 0.923, p = 0.757, p = 0.243), and (I) the number of photoreceptor nuclei above the OLM (p = 0.116, p = 0.034, p = 0.717, p = 0.730) in three *CRB1* patients and three isogenic control organoids with and without concomitant treatment of AAV.*GFP* pooled. Scale bars, 50 μm. Each datapoint in the graph represent individual organoids, of which an average has been taken of at least three representative images. The standard error of mean (SEM) is derived from these averages. Number of individual organoids per condition: Crb1 patients (P116, P117, and P128 pooled) treated with AAV.h*CRB1* (n = 34), AAV.h*CRB2* (n = 33), untreated and *GFP*-treated (n = 32), and isogenic controls (ISO-02 P116, ISO-03 P116, ISO-P128 pooled) treated with AAV.h*CRB1* (n = 14), AAV.h*CRB2* (n = 10), and untreated and *GFP*-treated (n = 24) independent organoid from two different differentiation rounds. Statistical tests: generalized linear mixed models with ^∗^p < 0.05, ^∗∗^p < 0.01, ^∗∗∗^p < 0.001. Related to Figure S4.

In addition, the long-term gene augmentation effect was examined for multiple *CRB1* patient-derived lines, where the organoids were collected and analyzed at DD210. One group of three different *CRB1* patient-derived retinal organoids was treated solely with AAV.h*CRB1* or AAV.h*CRB2* or left untreated, while in the following experiment the *CRB1* patient-derived retinal organoids were treated with AAV.h*CRB1* with AAV.*GFP*, or AAV.h*CRB2* with AAV.*GFP*, or AAV.*GFP* alone. Adding AAV.*GFP* facilitates in defining the regions where the AAV.h*CRB* most likely infected. Fluorescent images of organoids in culture co-treated with AAV.*GFP* and AAV.h*CRB* show the presence of GFP-positive regions, while no visible differences were observed between treated and untreated organoids using bright-field or fluorescent images (Figure 4A). Immunohistochemical staining of CRB1 in AAV.h*CRB1* or CRB2 in AAV.h*CRB2*-treated retinal organoids showed proof of recombinant CRB protein localization at the OLM and in the RPE (Figures 4B, 4C, and S4F–S4I). Further immunohistochemical analysis showed a partial improvement in the observed phenotype after AAV.h*CRB1* or AAV.h*CRB2* treatment (Figures 4D–4F). For quantitative analysis, all three *CRB1* patient-derived retinal organoids with and without concomitant treatment of AAV.*GFP* were pooled. No statistically significant difference was observed in fluorescence intensity of MUPP1 at the OLM in untreated compared with AAV.h*CRB*-treated *CRB1* patient-derived retinal organoids (Figure S4J). In addition, the expression of another core CRB-complex member, PALS1, is not changed after AAV.h*CRB* treatment (Figure S4K).

A statistically significant increased number of photoreceptor nuclei in a row was detected after AAV.h*CRB1* and AAV.h*CRB2* treatment at DD210, while this large difference was not observed in the treated isogenic controls (Figure 4G). Moreover, the ONL thickness (but not the retinal or the INL thickness) was significantly increased after AAV.h*CRB* treatment of *CRB1* patient-derived retinal organoids (Figures 4H, S4L, and S4M). Finally, the number of photoreceptor nuclei above the OLM was significantly improved after AAV.h*CRB2* treatment of *CRB1* patient-derived retinal organoids at DD210 (Figure 4I). No statistically significant improvement in the number of photoreceptor nuclei above the OLM was observed after AAV.h*CRB1* treatment of *CRB1* patient-derived retinal organoids nor after AAV.h*CRB1* or AAV.h*CRB2* treatment in the control retinal organoids (Figure 4I).

Altogether, these data show that the phenotype observed at DD180 and DD210 in *CRB1* patient-derived retinal organoids can be partially restored using AAV.h*CRB1* or AAV.h*CRB2* treatment.

### Differentially expressed genes related to the endosomal system are partially restored in AAV.h*CRB*-treated *CRB1* patient-derived retinal organoids

To identify gene expression changes upon *CRB* gene augmentation therapy, all three *CRB1* patient-derived retinal organoids were treated with AAV.h*CRB1* and AAV.*GFP* or AAV.h*CRB2* and AAV.*GFP* and compared with the AAV.*GFP*-treated control at DD230 using scRNA-seq. For all three patient-derived retinal organoids, we confirmed that the major retinal cell clusters were equally present in untreated and AAV-treated conditions (data not shown). A custom reference with the AAV.*GFP*, codon optimized AAV.h*CRB1*, and codon optimized AAV.h*CRB2* sequences was added to the dataset to detect which cell clusters were transduced. While analyzing all organoids and conditions together, we observed that AAV.*GFP* mainly transduces RPE, photoreceptor cells, transient I, and MGCs (Figure S5A). Specifically, 66% of the RPE, 35% of rods, 36% of cones, 37% of transient I, and 20% of MGCs contained AAV.*GFP* expression. This is in line with what we observed previously in the immunohistochemical analysis (Figures 3E and 3F). Next, AAV.h*CRB1* and AAV.h*CRB2* expression was analyzed in AAV.h*CRB*-treated retinal organoids. While being unable to fully distinguish exogenous and endogenous h*CRB* due to the high sequence similarity and the low levels of endogenous *CRB1* and *CRB2* in DD230 RPE, we observed a significant increase of AAV.h*CRB1* in AAV.h*CRB1*-treated and AAV.h*CRB2* in AAV.h*CRB2*-treated organoids in the RPE of the *CRB1* patient-derived retinal organoids (Figures S5B and S5C).

**Figure 5:**
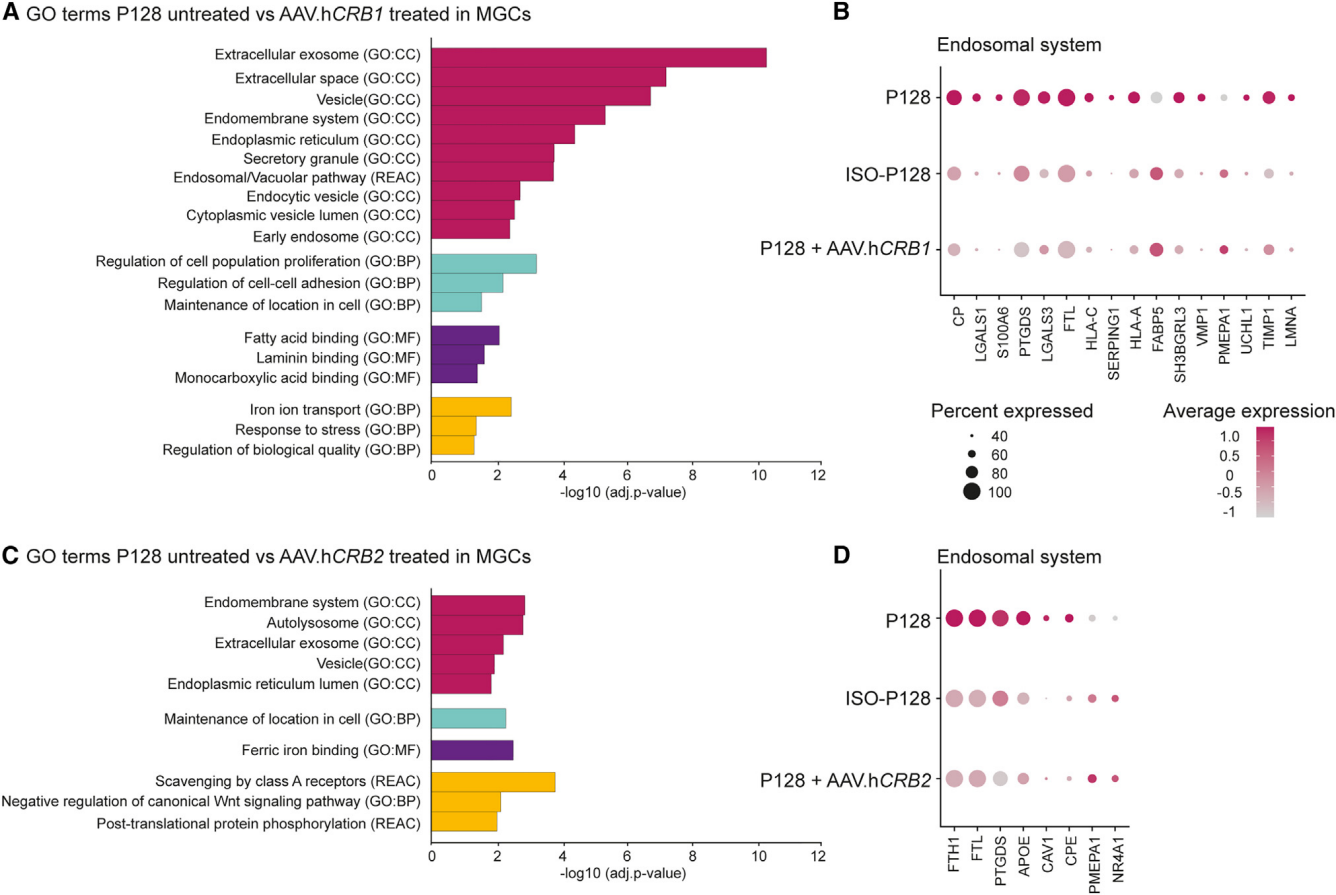
scRNA-seq of *CRB1* patient-derived retinal organoid treated with AAV.h*CRB1* or AAV.h*CRB2* restores transcriptional effect on the endosomal system (A and C) Gene ontology (GO) analysis of differentially expressed markers contrasting untreated with (A) AAV.h*CRB1-* or (C) AAV.h*CRB2*-treated P128 in MGCs clustered in groups with similar terms in the same color. (B and D) All significantly differentially expressed markers in terms related to the endosomal system after treatment with (B) AAV.h*CRB1* or (D) AAV.h*CRB2.* All markers present in (B and D) are also statistically significant for P128 compared with ISO-P128. Number of independent organoids used: P128, n = 6; P128+ AAV.h*CRB1*, n = 5; P128+ AAV.h*CRB2*, n = 5, from one differentiation round equally divided into three separate sequencing rounds. Related to Figure S5 and Table S5.

Differential gene expression followed by GO term analysis of AAV.h*CRB1* treatment compared with untreated P128 retinal organoids in MGCs revealed differences related to the endosomal system, cell-cell adhesion, and protein or receptor binding (Figure 5A). Also for AAV.h*CRB2* treatment similar terms were observed to be statistically significant in P128 (Figure 5C). Next, these GO terms and differentially expressed genes were compared with the ones observed when contrasting P128 with ISO-P128. Overlapping differentially expressed genes associated with the endosomal system show that, after AAV.h*CRB1* treatment, the expression levels from the patient retinal organoids are similar to levels of the isogenic control (Figure 5B). Moreover, after AAV.h*CRB2* treatment the genes associated with the endosomal system appeared to be restored as well in MGCs (Figure 5D).

Similar comparisons were performed for the other two *CRB1* patient-derived retinal organoids. For P116 and P117, statistically significant GO terms related to the endosomal system were also observed after AAV.h*CRB1* and AAV.h*CRB2* treatment (Figures S5D, S5E, S5G, S5I, and S5K). When analyzing in more detail the differentially expressed genes associated with the endosomal system in AAV.h*CRB1*-treated P116 organoids, transcript levels seemed to be restored to isogenic control levels in MGCs (Figure S5J) as well as in rod photoreceptor cells (Figure S5L). The genes for ISO-P116, indicated with a dashed line box around them, were not statistically significantly different from P116, but the genes after AAV.h*CRB* treatment are similarly expressed as the average expression in ISO-P116 (Figure S5J). Similar results were observed for genes associated with the endosomal system in AAV.h*CRB2*-treated organoids (Figure S5I). Moreover, after both AAV.h*CRB1* and AAV.h*CRB2* treatment in MGCs of P117, we observed that the expression levels changed in a similar direction (Figure S5F).

In summary, AAV.h*CRB1* as well as AAV.h*CRB2* treatment on *CRB1* patient-derived retinal organoids restores gene expression related to the endosomal system back to isogenic control levels.

## Discussion

In this manuscript we (1) showed diminished levels of variant CRB1 protein in *CRB1* RP patient retinal organoids that harbor missense mutations, (2) demonstrate moderate loss of photoreceptors in *CRB1* patient-derived retinal organoids at DD210, (3) detect transcriptional differences suggesting changes within the endosomal system in *CRB1* patient compared with isogenic control organoids, (4) show that AAV5.CMV.*GFP* efficiently transduced MGCs in addition to photoreceptors and RPE at DD120, and (5) observe a partially restored phenotype after AAV.h*CRB1-* or AAV.h*CRB2*-mediated gene therapy in *CRB1* patient-derived retinal organoids.

*CRB1* patients’ clinical and genetic characteristics were described previously in detail in a prospective natural history study on 22 patients (Nguyen et al., 2022). The P116 retinal organoids were derived from skin fibroblasts from a patient with a first diagnosis RP and discontinuous OLM and ellipsoid zone (EZ) in parafovea and perifovea on spectral domain optical coherence tomography (see Tables S1 and S2 in Nguyen et al. [2022]). The P117 retinal organoids were derived from a patient who experienced mild RP with at first diagnosis loss of visual acuity and continuous OLM and EZ in parafovea and perifovea. The P128 retinal organoids were derived from a patient with at first diagnosis RP, nyctalopia, and discontinuous OLM and EZ in parafovea and perifovea. Here, the *CRB1* patient-derived retinal organoids were compared with corresponding isogenic controls at DD210. We show a decreased number of photoreceptor nuclei in a row and a reduced ONL thickness in the *CRB1* patient-derived retinal organoids compared with the isogenic controls. Decreased levels of variant CRB1 at the OLM of *CRB1* patient-derived retinal organoids might be associated with increased protrusion of photoreceptor cell bodies into the cell culture medium at DD180 (Quinn et al., 2019b) and thinning of the photoreceptor ONL at DD210 (current article). This process is similar to the complete loss of CRB1 at the OLM in *Crb1* mouse retina which results in protrusion of photoreceptor cell bodies into the subretinal space (van de Pavert et al., 2004, van de Pavert et al., 2007a, van de Pavert et al., 2007b) or the loss of CRB2 at the OLM in *Crb2* mouse retina (Alves et al., 2013).

Moreover, it was shown that CRB1 and CRB2 are present at the OLM of both photoreceptor and MGCs in iPSC-derived retinal organoids (Quinn et al., 2019b). Our scRNA-seq data confirm on the transcriptome, with more *CRB1* expression in MGCs than in photoreceptor cells and more *CRB2* expression in photoreceptor cells than in MGCs. The rather high expression levels of *CRB1* and low levels of *CRB2* in MGCs might be related to the phenotype variation, since mutations in *CRB1* may cause either early onset RP or LCA. Variable low levels of *CRB2* transcripts in MGCs of *CRB1* patients may be involved in the severity of the phenotype. Such a hypothesis would be in strong correlation with our previous studies in mice, which suggests a modifying role for *CRB2* in *CRB1*-related dystrophies (Buck et al., 2021; Pellissier et al., 2014b, 2015; Quinn et al., 2018, 2019a).

CRB1 variant protein at the OLM was strongly diminished in *CRB1* patient-derived retinal organoids, while *CRB1* expression levels remained similar. In contrast, CRB core complex members as well as the FERM proteins remained at the OLM and similar expression levels were observed. This indicates that a variant CRB1 protein is produced but it does not localize to or maintain its expected location at the OLM. The endolysomal system is required for transport of CRB1 to the OLM but also for recycling of endocytosed CRB1 from the early endosome to the OLM and the transport into degradative vesicles. *Drosophila* studies show that CRB trafficking is mediated by transport along microtubules by Rab11- and retromer-containing endosomes (Aguilar-Aragon et al., 2020; Pocha et al., 2011). In addition, in *Drosophila* salivary gland cells Crumbs maintains the active pool of Rab proteins at the apical domain, which is essential for maintaining the organization of the apical membrane and efficient apical secretion (Lattner et al., 2019). The scRNA-seq GO data shown here suggest an aberrant endosomal pathway specifically in MGCs and rods of *CRB1* patient-derived retinal organoids. Dysregulation of CRB1 at the OLM can thus cause changes in the endosomal system. Endosomal recycling is pivotal for maintenance of neuronal health, and defects in its function results in human neurodegenerative disorders (Saitoh, 2022; Tang et al., 2015). We hypothesize that the reduced levels of variant CRB1 at the OLM are caused by disturbed variant CRB1 protein transport to the OLM, or disturbed endosomal recycling of variant CRB1 between OLM and the early endosome, or increased variant CRB1 degradation in the retinal organoids. Preliminary studies suggest changes in the recycling endosome and in degradative vesicles (T.M.B. and J.W., unpublished data). In analogy to the roles of the Crumbs protein in *Drosophila* salivary glands (Lattner et al., 2019), in future studies we will examine the putative role for CRB1 in the maintenance of an active pool of RAB11 and VPS35 (retromer) recycling endosome proteins at the OLM.

Previously, we described an improved phenotype after AAV.h*CRB2* treatment in *Crb* mutant mouse models (Buck et al., 2021; Pellissier et al., 2015). Here, we investigated whether we could observe an improved *CRB1*-RP phenotype after AAV-mediated gene augmentation therapy in *CRB1* patient-derived retinal organoids. Proof-of-concept for developing gene therapy in retinal organoids for *CRX*-LCA has been described, where AAVs were used to alleviate the phenotype observed in *CRX* mutant retinal organoids (Kruczek et al., 2021). In addition, AAV-mediated gene augmentation of RP2 knockout retinal organoids prevents ONL thinning and restored rhodopsin expression (Lane et al., 2020). In this article, using AAV.h*CRB1* and AAV.h*CRB2* gene augmentation therapy, a partially restored phenotype was observed in *CRB1* patient-derived retinal organoids. The number of photoreceptor nuclei in a row and ONL thickness were significantly improved after AAV.h*CRB* treatment when analyzed at DD210, showing the long-term effects of the gene augmentation therapy. Moreover, neither positive nor negative effects were observed when treating isogenic controls with AAV.h*CRB*1 or AAV.h*CRB2*. Furthermore, the infection of AAV.h*CRB1* on the *CRB1*^KO^ retinal organoids shows localization of recombinant CRB1 protein at the OLM. Whereas the recombinant CRB1 protein localizes merely at the OLM, we also detected CRB1 protein around the OLM as detected previously in first trimester human fetal retina and DD120 immature wild-type retinal organoids (Quinn et al., 2019b). The imprecise localization of recombinant CRB1 is potentially related to a partial restoration of CRB1-positive recycling endosomal vesicles at the OLM and is the subject of future studies. To our knowledge, this is the first time that an improved phenotype after AAV.h*CRB* gene augmentation in *CRB1* patient-derived retinal organoids has been observed.

In conclusion, we demonstrate in *CRB1* patient-derived retinal organoids a moderate loss of photoreceptor nuclei in a row, strongly reduced levels of CRB1 variant protein with unaffected *CRB1* transcript levels, and a dysregulated molecular gene profiling phenotype of MGCs and rod photoreceptor cells, suggesting an aberrant endosomal system. Moreover, using AAV-mediated gene augmentation therapy approaches we have improved the histological and transcriptional retinal phenotype in *CRB1* patient-derived retinal organoids. These data provide essential information for future gene therapy approaches for patients with mutations in the *CRB1* gene.

## Experimental procedures

### Resource availability

#### Corresponding author

Further information and requests for resources and reagents should be directed to and will be fulfilled by the corresponding author, Jan Wijnholds (j.wijnholds@lumc.nl).

#### Materials availability

Materials and additional details can be made available from the corresponding author upon reasonable request.

### Cell culture and retinal organoid differentiation

The following hiPSC lines were used for organoid differentiation: three *CRB1* RP patient-derived lines and one control (LUMC0116iCRB09, LUMC0117iCRB01, LUMC0128iCRB01, LUMC0004iCTRL10 [Quinn et al., 2019b]), and three isogenic controls of the *CRB1* patient-derived lines (LUMC0116iCRB-ISO02, LUMC0116iCRB-ISO03, LUMC0128iCRB-ISO01) (Figure S1; Table S1). hiPSC lines were derived from skin fibroblast using polycistronic lentiviral vectors (Warlich et al., 2011).

hiPSCs were maintained on Matrigel-coated plates in mTeSR plus medium and passaged mechanically using gentle cell dissociation reagent (STEMCELL Technologies). Retinal organoid differentiation was carried out as reported previously with some modifications (experimental procedures in supplemental information) (Quinn et al., 2019b; Zhong et al., 2014). Retinal organoids were collected at DD180 or DD210 for immunohistochemical analysis; a list of all primary antibodies used for immunofluorescent staining is provided in (Table S2). At least three different differentiation batches were analyzed to verify disease phenotypes.

### AAV transduction of hiPSC-derived retinal organoids

Two to three retinal organoids were plated in a 96-well agarose-coated plate and were infected with AAV in 50 μL RLM2 and incubated for 8 h at 5% CO_2_ at 37°C. After this, the wells were topped up to 200 μL with RLM2. The next morning, treated organoids were transferred to a 24-well plate and cultured for at least 3 weeks or until the desired differentiation day. AAV5.CMV.*GFP* and AAV2.CMV.*GFP* (105530; Addgene) were used at titers of 1 × 10^10^, 3.3 × 10^10^, 6.6 × 10^10^, or 10 × 10^10^ gc. AAV5.CMVmin.h*CRB1* and AAV5.CMV.h*CRB2* (HORAMA) were used at a titer of 3.3 × 10^10^ gc.

### Quantification and statistical analysis

Magnification images (40×) were manually quantified using Fiji ImageJ (ImageJ 1.53f51). At least four organoids per condition with three to six representative images of each organoid were used for quantification. Three regions in each image were manually analyzed for the number of photoreceptor nuclei in a row in the ONL, the number of photoreceptor nuclei above the OLM, retinal thickness, INL thickness, and ONL thickness. Quantifications were performed independently by at least two researchers without the knowledge of genotype or treatment. For the MUPP1 quantifications, a ROI was drawn at the OLM and the average intensity was measured using ImageJ. All datapoints measured were averaged per organoid and plotted in the graph; so that each point is one organoid. No normalization of the values was performed. Data were either presented per 100 μm retinal length or per field of view. Data presentation and statistical analysis were performed using GraphPad Prism version 8 (GraphPad Software) and IBM SPSS statistics (version 25), respectively. For statistical analysis, all individual values per image were used. A generalized linear mixed model with treatment (and patient) as a fixed effect was performed on all quantification parameters; the statistical test took into account that multiple *CRB1* patients were merged by introducing a random intercept per patient. Data are presented as mean per organoid ± standard error of the mean. Significance is indicated in graphs as ^∗^p < 0.05, ^∗∗^p < 0.01, and ^∗∗∗^p < 0.001.

### scRNA-seq

Retinal organoids were dissociated using an adapted protocol from the Papain Dissociation Kit (Worthington, I-LK 03150). Analysis and processing of single-cell transcriptomics using Seurat is detailed in experimental procedures in supplemental information.

## Author contributions

Conceptualization, N.B. and J.W.; software, N.B., I.M., S.B., and H.M.; formal analysis, N.B.; investigation, N.B., X.L., C.A.A., and T.M.B.; resources, C.F. and C.H.A.; data curation, N.B. and I.M.; writing – original draft, N.B.; writing – review & editing, N.B. and J.W.; visualization, N.B.; project administration, J.W.; funding acquisition, J.W.

## Data Availability

scRNA-seq data are available at the NCBI Gene Expression Omnibus database (GEO: GSE212582)*.*

## Supplemental information

Document S1. Figures S1–S5, Tables S1 and S2, and supplemental experimental procedures

Table S3. Top differentially expressed genes per cluster, related to Figure 2 Statistically significant log2 fold changes of the expression level of differentially expressed genes in the cluster (pct1) compared with the remaining clusters (pct2). With the defined cell type per cluster.

Table S4. Differentially expressed gene markers and gene ontology terms contrasting P128 and ISO-P128, and P116 and ISO-P116, related to Figures 2 and S3A–S3E Log2 fold changes of the expression level of statistically significant expressed genes in CRB1 patient-derived retinal organoids compared with the isogenic control in MGCs or in rod photoreceptor cells and the associated gene ontology terms.

Table S5. DEG markers and gene ontology terms comparing untreated and AAV.hCRB-treated CRB1 patient-derived retinal organoids, related to Figures 5 and S5 Log2 fold changes of the expression level of statistically significant expressed genes in AAV.hCRB-treated CRB1 patient-derived retinal organoids compared with the untreated and the associated gene ontology term.

Document S2. Article plus supplemental information
