# AAV-mediated gene augmentation therapy of *CRB1* patient-derived retinal organoids restores the histological and transcriptional retinal phenotype

**DOI:** 10.1016/j.stemcr.2023.05.008

**Published:** 2023-06-13

**Authors:** Nanda Boon, Xuefei Lu, Charlotte A. Andriessen, Ioannis Moustakas, Thilo M. Buck, Christian Freund, Christiaan H. Arendzen, Stefan Böhringer, Camiel J.F. Boon, Hailiang Mei, Jan Wijnholds

## Main text

(Stem Cell Reports *18*, 1123–1137; May 9, 2023)

In the originally published manuscript by Boon N. et al (“AAV-mediated gene augmentation therapy of CRB1 patient-derived retinal organoids restores the histological and transcriptional retinal phenotype”), the first and last authors neglected to add the coauthor Camiel J.F. Boon. During the revision of the manuscript, the addition of C.J.F. Boon was discussed in writing between the first and last author, but due to a misunderstanding, C.J.F. Boon was not added as a coauthor. C.J.F. Boon made significant contributions to the revised manuscript by being responsible for patient materials and information, and C.J.F. Boon has now been added to the author list in the online version of the manuscript. Note that there are no familial or other relationships between C.J.F. Boon and N. Boon.

Additionally, in the originally published version of the supplemental figures that accompanied our manuscript, the majority of the legend for Figure S2 was accidentally removed. We have since corrected the supplemental figures and the complete legend is now contained therein. We apologize for any confusion.

