# Supplementary material for: AAV-mediated gene augmentation therapy of CRB1 patient-derived retinal organoids restores the histological and transcriptional retinal phenotype
Source: Stem Cell Reports. 2023 Apr 20;18(5):1123–37. doi: 10.1016/j.stemcr.2023.03.014 (PMC10202653; doi:10.1016/j.stemcr.2023.03.014)
Supplement: Document S1. Figures S1–S5, Tables S1 and S2, and supplemental experimental procedures [file mmc1.pdf]

Stem Cell Reports, Volume *18*

Supplemental Information

AAV-mediated gene augmentation therapy of *CRB1* patient-derived retinal organoids restores the histological and transcriptional retinal phenotype

Nanda Boon, Xuefei Lu, Charlotte A. Andriessen, Ioannis Moustakas, Thilo M. Buck, Christian Freund, Christiaan H. Arendzen, Stefan Böhringer, Hailiang Mei, and Jan Wijnholds

Supplemental Figures and Legends

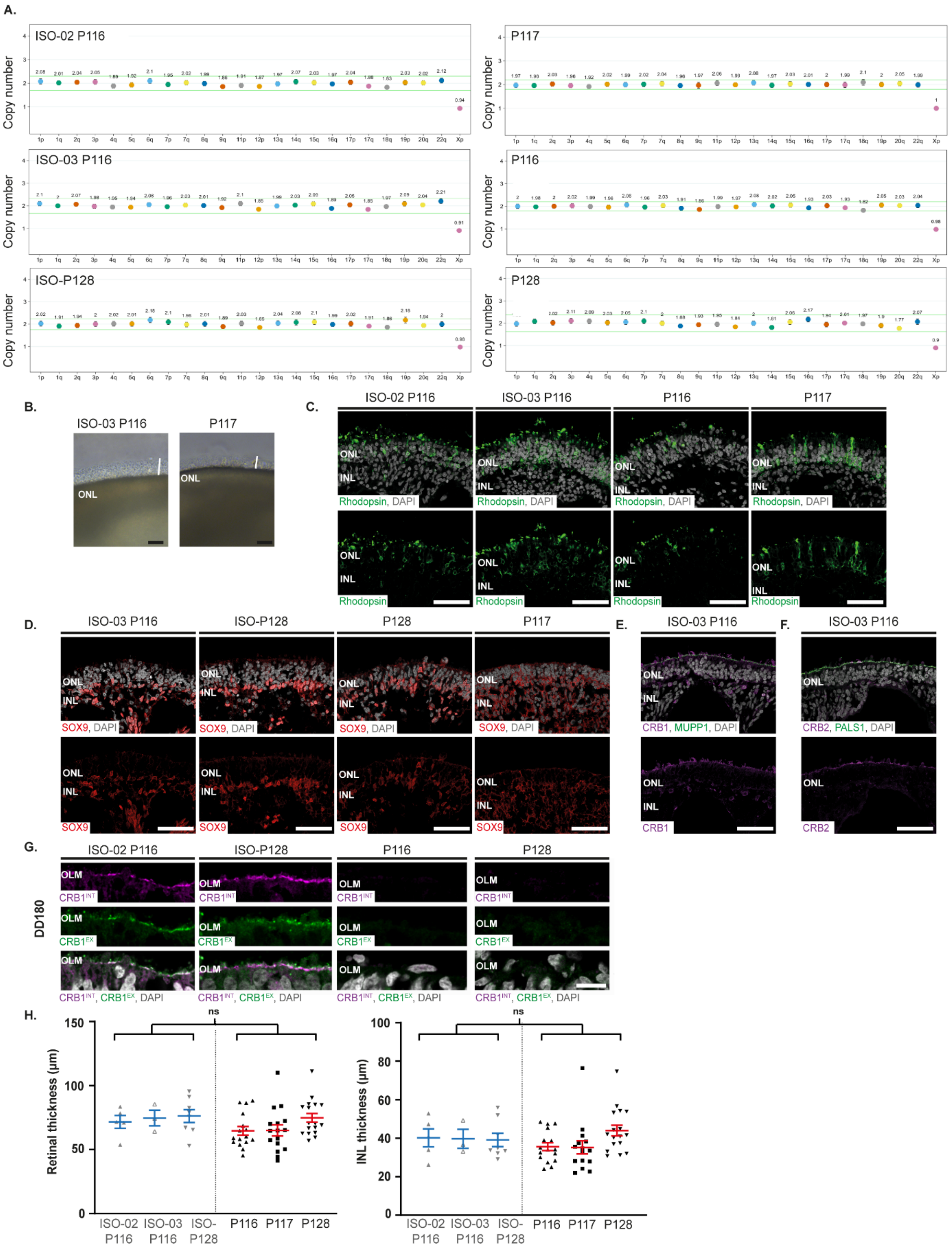

Figure S1: *CRB1* patient derived and isogenic control retinal organoids phenotypic analysis at DD210. Related to Figure 1. (A) Meta-analysis of 90% most recurrent abnormalities in hiPSCs showing no abnormalities after creating the isogenic controls used in this study. (B) Representative brightfield images of ISO-03 P116 and P117 cultured organoids. (C) Representative immunohistochemical images of rhodopsin (green) in ISO-02 P116, ISO-03 P116, P116, and P117. (D) Representative immunohistochemical images of SOX9 (red) in ISO-P128, P128, P117, and ISO-

03 P116. (E, F) Representative immunohistochemical images of (E) CRB1 (magenta) co-localized with MUPP1 (green) and of (F) CRB2 (magenta) co-localized with PALS1 (green) in ISO-03 P116. (G) Representative immunohistochemical images of $CRB1^{EX}$ (green) and $CRB1^{INT}$ (magenta) in *CRB1* patient-derived retinal organoids compared to isogenic controls at DD180. (H) Quantitative analysis of the total retinal thickness (p=0.158) and INL thickness (p=0.696) per field of view in *CRB1* patient derived and isogenic control retinal organoids. Each datapoint in the graph represent individual organoids, of which an average has been taken of at least 3 representative images. The standard error of mean (SEM) is derived from these averages. Number of individual organoids per condition and differentiation round: P116 *n*=16, P117 *n*=15, P128 *n=17* from four independent organoid batches, ISO-P128 *n=8* from three independent organoid batches, ISO-02 P116 *n*=5 and ISO-03 P116 *n=5* from two independent organoid batches. Scalebar = (C-F) 50µm, (G) 10µm.

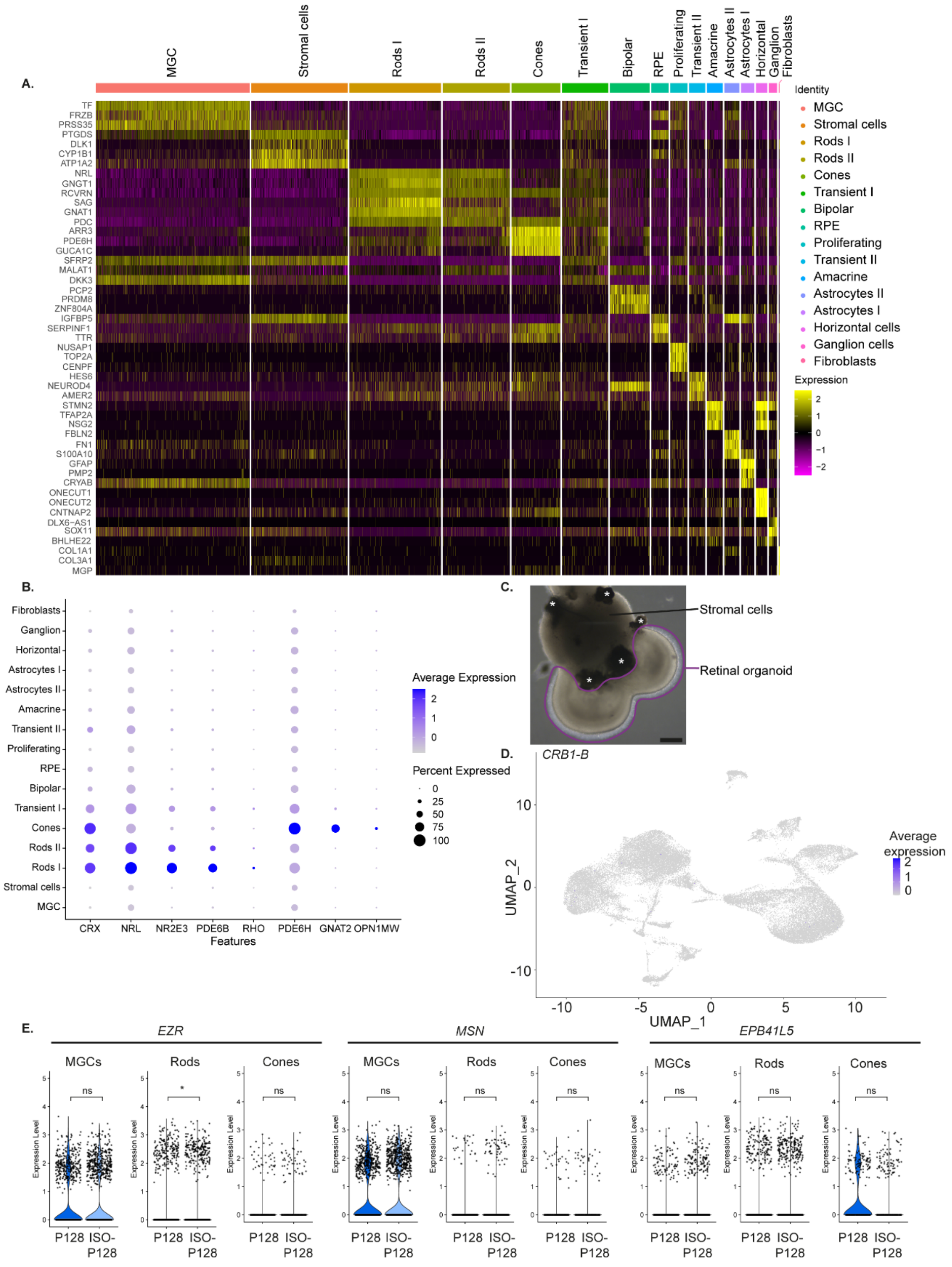

A.
MGC
Stromal cells
Rods I
Rods II
Cones
Transient I
Bipolar
RPE
Proliferating
Transient II
Amacrine
Astrocytes II
Astrocytes I
Horizontal
Ganglion
Fibroblasts
TF
FRZB
PRSS35
PTGDS
DLK1
CYP1B1
ATP1A2
NRL
GNGT1
RCVRN
SAG
GNAT1
PDC
ARR3
PDE6H
GUCA1C
SFRP2
MALAT1
DKK3
PCP2
PRDM8
ZNF804A
IGFBP5
SERPINF1
TTR
NUSAP1
TOP2A
CENPF
HES6
NEUROD4
AMER2
STMN2
TFAP2A
NSG2
FBLN2
FN1
S100A10
GFAP
PMP2
CRYAB
ONECUT1
ONECUT2
CNTNAP2
DLX6−AS1
SOX11
BHLHE22
COL1A1
COL3A1
MGP
Identity
MGC
Stromal cells
Rods I
Rods II
Cones
Transient I
Bipolar
RPE
Proliferating
Transient II
Amacrine
Astrocytes II
Astrocytes I
Horizontal cells
Ganglion cells
Fibroblasts
Expression
2
1
0
−1
−2
B.
Fibroblasts
Ganglion
Horizontal
Astrocytes I
Astrocytes II
Amacrine
Transient II
Proliferating
RPE
Bipolar
Transient I
Cones
Rods II
Rods I
Stromal cells
MGC
CRX
NRL
NR2E3
PDE6B
RHO
PDE6H
GNAT2
OPN1MW
Features
Average Expression
2
1
0
Percent Expressed
0
25
50
75
100
C.
Stromal cells
Retinal organoid
D.
CRB1-B
10
0
-10
UMAP_2
-10
-5
0
5
10
UMAP_1
Average expression
2
1
0
E.
EZR
MSN
EPB41L5
MGCs
Rods
Cones
ns
*
Expression Level
P128
ISO-P128

p=0.41, p=0.17, p=0.59), rods (p=0.018, p=0.89, p=0.53) and cones (p=0.77, p=0.79, p=0.24) in P128 vs ISO-P128. Number of independent organoids used: ISO-P128 *n*=6, and P128 *n*=6 from one differentiation round equally divided into three separate sequencing rounds.

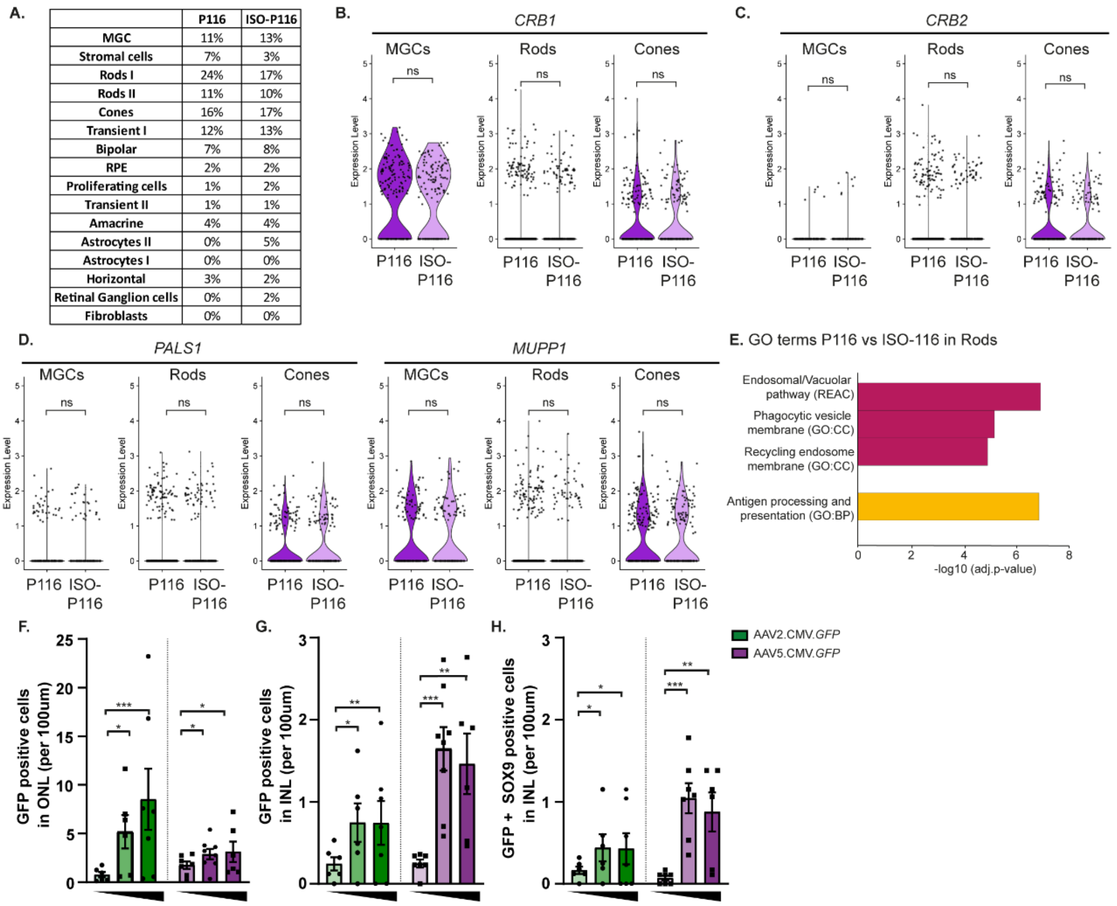

A.

	P116	ISO-P116
MGC	11%	13%
Stromal cells	7%	3%
Rods I	24%	17%
Rods II	11%	10%
Cones	16%	17%
Transient I	12%	13%
Bipolar	7%	8%
RPE	2%	2%
Proliferating cells	1%	2%
Transient II	1%	1%
Amacrine	4%	4%
Astrocytes II	0%	5%
Astrocytes I	0%	0%
Horizontal	3%	2%
Retinal Ganglion cells	0%	2%
Fibroblasts	0%	0%

Figure S3: scRNA-seq analysis comparing ISO-P116 and P116 retinal organoids. Related to Figure 2. (A) Table showing all retinal cell types equally present in the retinal organoids. (B) Violin plots of CRB1 and (C) CRB2 expression levels specifically in MGCs (p=0.78, p=0.28), rods (p=0.37, p=0.88), and cones (p=0.51, p=0.96). (D) Violin plots of canonical core CRB complex members PALS1, and MUPP1 levels in (p=0.49, p=0.98), rods (p=0.36, p=0.61), and cones (p=0.41, p=0.86). (E) Gene ontology (GO) analysis of differentially expressed markers specifically in rods clustered with similar terms in the same colour. Number of independent organoids used: ISO-P116 *n*=4, and P116 *n*=4 from one differentiation and sequencing round. (F, G, H) Quantification of AAV2.CMV.*GFP* and AAV5.CMV.*GFP* retinal organoids at DD120 with three different titre concentrations: $1x10^{10}$, $6.6x10^{10}$, and $10x10^{10}$ gc (genome copies) in the (F) ONL, (G) INL, and (H)) GFP positive MGC in the INL per 100µm. Each datapoint in the graph represent individual organoids, of which an average has been taken of at least 3 representative images. The standard error of mean (SEM) is derived from these averages. Number of organoids per condition: for AAV2.CMV.*GFP* $1x10^{10}$ n=5, $6.6x10^{10}$ n=6, and $10x10^{10}$ n=7, and for AAV5.CMV.*GFP* $1x10^{10}$ n=7, $6.6x10^{10}$ n=8, and $10x10^{10}$ n=6 individual organoids from two independent differentiation rounds. Statical analysis: generalized linear mixed models with p<0.05 (*), p<0.01 (**), and p<0.001 (***).

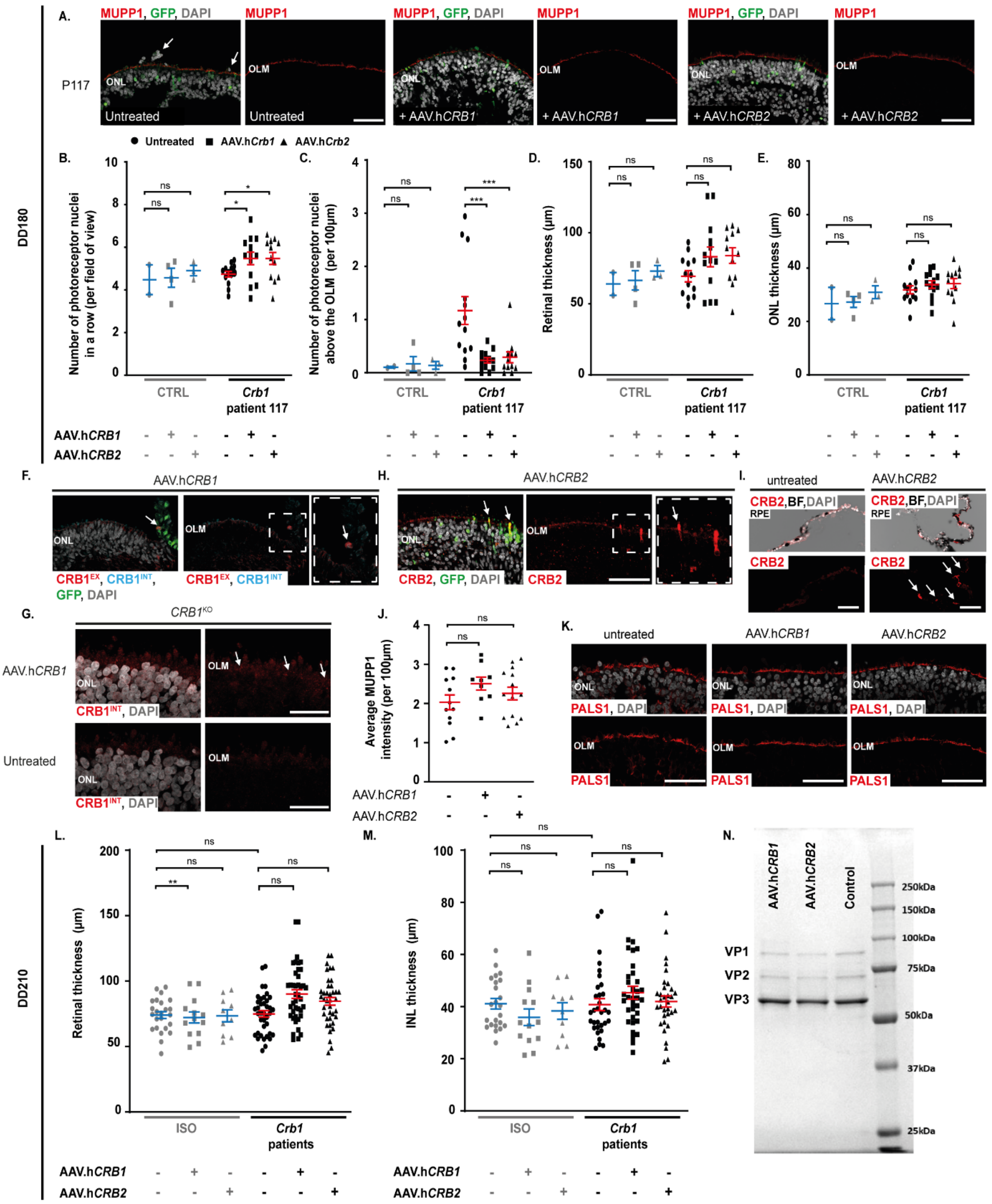

Figure S4: AAV-mediated gene therapy treatment on *CRB1* patient-derived and isogenic control retinal organoids. Related to Figure 4. (A) Representative immunohistochemical images of untreated, AAV.h*CRB1*, and AAV.h*CRB2* treated P117 and control retinal organoids stained with MUPP1 (red) and AAV.*GFP* (green) at DD180. (B) Quantification at DD180 of the number of photoreceptor nuclei in a row per field of view (from left to right: *p*=0.042, *p*=0.041, *p*=0.925, *p*=0.548), (C) the number of photoceptor nuclei above the OLM per 100 µm (*p*=0.000, *p*=0.001, *p*=0.676, *p*=0.865), (D) retinal thickness per field of view (*p*=0.104, *p*=0.078, *p*=0.819, *p*=0.389), and (E) ONL thickness per field of view (*p*=0.438, *p*=0.355, *p*=0.933, *p*=0.378). (F) Representative

immunohistochemical images of anti-CRB1^{EX} and anti-CRB1^{INT} showing localization in the RPE after AAV.h*CRB1* treatment in *CRB1* patient-derived retinal organoid. (G) Immunohistochemical image of anti-CRB1^{INT} showing localization in the OLM after AAV.h*CRB1* treatment in *CRB1*^{KO} retinal organoid. (H, I) Representative immunohistochemical images of CRB2 at the OLM (H) and RPE (I) after AAV.h*CRB2* treatment in *CRB1* patient-derived retinal organoids. (J) Quantification of the average MUPP1 fluorescence intensity at the OLM of DD210 *CRB1* patient derived retinal organoids (P116, P117, P128 pooled) treated with AAV.h*CRB*. (K) Representative immunohistochemical images of PALS1 at the OLM with and without AAV.h*CRB* treatment in a *CRB1* patient-derived retinal organoid. (L) Quantification of the retinal thickness (p=0.008, p=0.082, p=0.993, p=0.981, p=0.981) and (M) the INL thickness (p=0.139, p=0.632, p=0.958, p=0.195, p=0.707) per field of view of *CRB1* patient and isogenic control retinal organoids at DD210. (N) SDS-PAGE gel of AAV.h*CRB1* and AAV.h*CRB2* showing no contamination in the AAV preparation. Scalebar = 50µm. Each datapoint in the graph represent individual organoids, of which an average has been taken of at least 3 representative images. The standard error of mean (SEM) is derived from these averages. Number of individual organoids per condition at DD180 P117 treated with AAV.*hCRB1 n=13,* AAV.h*CRB2 n*=12, untreated *n*=14 from two different differentiation rounds, control organoids treated with AAV.h*CRB1 n=4*, AAV.h*CRB2 n*=3, and untreated *n*=2 from one differentiation round. And at DD210 *CRB1* patient-derived retinal organoids (P116, P117, P128 pooled) treated with AAV.*hCRB1 n*=34*,* AAV.h*CRB2* *n*=33, untreated *n*=32, and isogenic controls (ISO-02 P116, ISO-03 P116, ISO-P128 pooled) treated with AAV.h*CRB1 n*=14, AAV.h*CRB2 n*=10, and untreated *n*=24 independent organoid from two different differentiation rounds. Statistical tests: generalized linear mixed models with $p<0.05$ (*), $p<0.01$ (**), and $p<0.001$ (***).

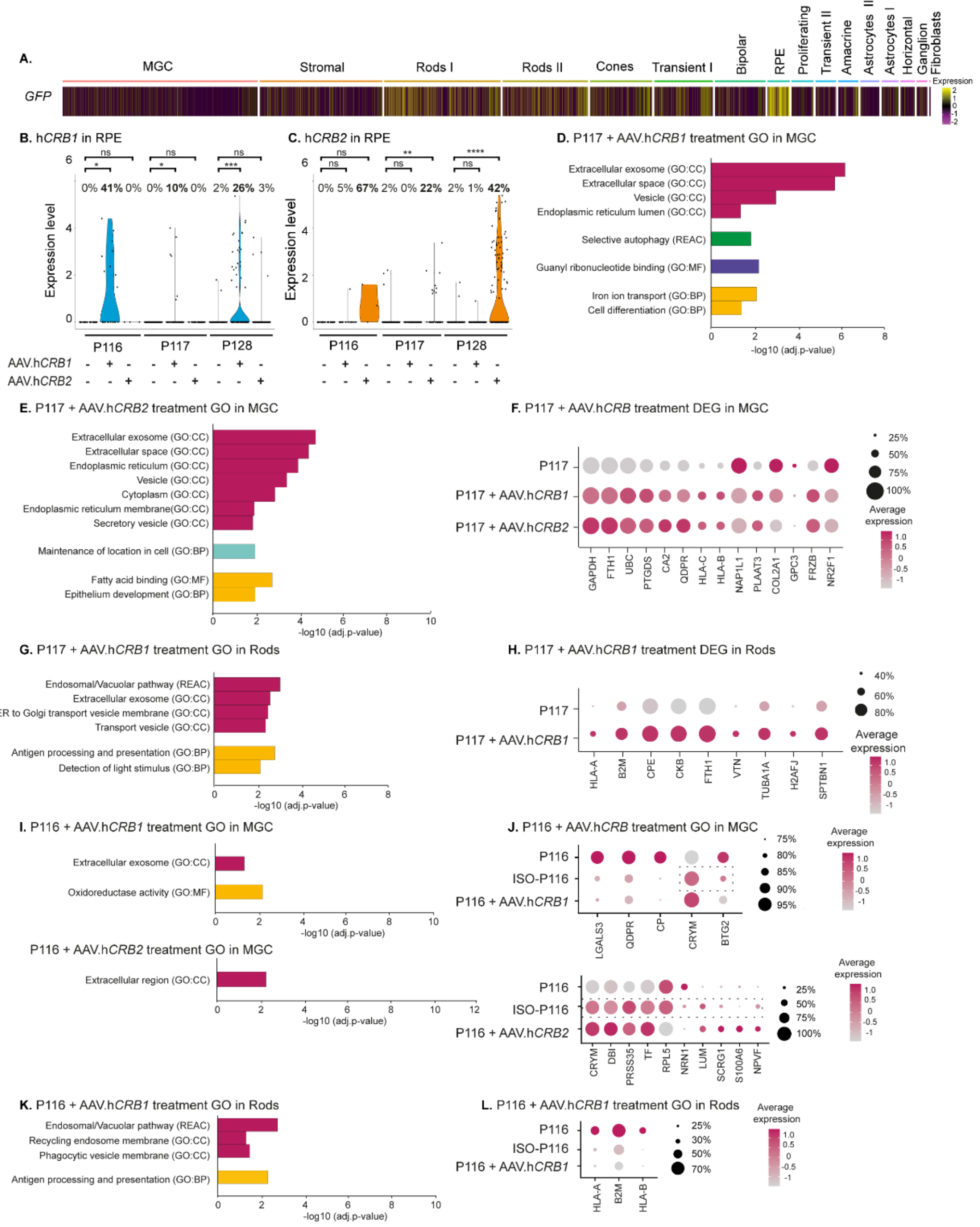

Figure S5: Single cell RNA-sequencing of *CRB1* patient-derived retinal organoid treated with AAV.h*CRB1* or AAV.h*CRB2* restores transcriptional effect on the endosomal system. Related to Figure 5. (A) Heatmap showing AAV.*GFP* expression in the clusters, mainly transducing MGC, photoreceptor cells, and RPE. (B, C) Violin plot of transcript expression of (B) AAV.h*CRB1* (from left to right: p=0.018, p=0.41, p=0.041, p=0.078, p<0.001, p=0.54) or (C) AAV.h*CRB2* (p=0.5, p=0.1, p=0.38, p=0.0037, p=0.53, p<0.00001) in the RPE of AAV.h*CRB* treated retinal organoids. (D, E) Gene ontology (GO) analysis of differentially expressed markers contrasting untreated with (D) AAV.h*CRB1* or (E) AAV.h*CRB2* treated P117 in MGCs clustered in groups with similar terms in the same colour. (F) All significantly differentially expressed markers in terms related to the endosomal system after treatment with AAV.h*CRB1* orAAV.h*CRB2* in MGC. (G, H) P117 treated with AAV.h*CRB1* in rods showing gene ontology (G) and all terms related to the endosomal system (H). (I)

GO of P116 treatment with AAV.h*CRB* in MGC and (J) dot plot of terms related to the endosomal system after treatment. The data in the box with a dashed line are not statistically significant different from P116, all the other data points (in F, H, J, and L) are statistically significant different from untreated patient derived retinal organoids. Number of independent organoids used: P116 *n*=4, P116 + AAV.h*CRB1* *n*=3, P116 + AAV.h*CRB2* *n*=3 from one differentiation and sequencing round, and P117 *n*=5, P117 + AAV.h*CRB1* *n*=5, P117 + AAV.h*CRB2* *n*=5 from one differentiation round equally divided into three separate sequencing rounds.

Supplemental Tables

Table S1. hiPSC line information. Related to all figures.

Previously published line name	Description	Gender
LUMC04iCTRL10 (Quinn et al., 2019)	Control iPSC line	Male
CRB1^{KO}LUMC04iCTRL10	*CRB1*^{KO} line was derived from LUMC04iCTRL10; it has a stop codon in the second exon of *CRB1* (Boon N et al Wijnholds J, unpublished data). Only used for proof of recombinant CRB1 protein expression after AAV.h*CRB1* treatment.	Male
LUMC0116iCRB09 (Quinn et al., 2019)	P116 = Allele 1 and 2: homozygous c.3122T>C--> p.(Met1041Thr)	Male
iso02LUMC0116iCRB09	ISO-02 P116 = Allele 1: c.3122T>C gene corrected to c.3120C>G. Allele 2: c.3122T>C. p.(Met1041Thr)	Male
iso03LUMC0116iCRB09	ISO-03 P116 = Homozygous c.3122T>C gene corrected to c.3120C>G	Male
LUMC0117iCRB01 (Quinn et al., 2019)	P117 = Allele 1: c.1892A>G (p.Tyr631Cys)). Allele 2: c.2911G>T (p.(Glu995*)	Male
LUMC0128iCRB01 (Quinn et al., 2019)	P128 = Allele 1: c.2843G>A --> p.(Cys948Tyr). Allele 2: c.3122T>C --> p.(Met1041Thr)	Male
iso02LUMC0128iCRB01	ISO-P128 = Allele 1: c.2843G>A. p.(Cys948Tyr). Allele 2: c.3122T>C gene corrected to c.3120C>G	Male

Table S2. List of primary antibodies used in the study. Related to all figures.

Antigen	Dilution	Source	Identifier
CRB1 (intracellular domain – used if not otherwise specified)	1:200	Homemade	NA
CRB1 (extracellular domain)	1:200	Abnova	H00023418-A01
CRB2	1:200	Homemade	NA
CRALBP	1:200	Abcam	ab15051
MUPP1	1:200	BD Biosciences	M98820

PALS1	1:200	Homemade	NA
OTX2	1:200	Proteintech	13497-1-AP
Rhodopsin	1:200	Sigma	SAB4502636
Glutamine synthetase (GS)	1:250	BD Biosciences	610518
SOX9	1:250	Millipore	AB5535

Table S3. Top differentially expressed genes per cluster. Related to Figure 2. Statistically significant log2 fold changes of the expression level of differentially expressed genes in the cluster (pct1) comparing with the remaining clusters (pct2). With the defined cell type per cluster.

Table S4. Differentially expressed genes markers and gene ontology terms contrasting P128 and ISO-P128, and P116 and ISO-P116. Related to Figure 2, S3A-E. Log2 fold changes of the expression level of statistically significant expressed genes in *CRB1* patient-derived retinal organoids comparing with the isogenic control in MGCs or in rod photoreceptor cells and the associated gene ontology terms.

Table S5. DEG markers and gene ontology terms comparing untreated and AAV.h*CRB* treated *CRB1* patient derived retinal organoids. Related to Figure 5, S5. Log2 fold changes of the expression level of statistically significant expressed genes in AAV.h*CRB* treated *CRB1* patient-derived retinal organoids comparing with the untreated and the associated gene ontology terms.

Supplemental experimental procedures

Cell culture and retinal organoid differentiation

Human induced pluripotent stem cells (hiPSC) were maintained on Matrigel coated plates in mTeSR plus medium (STEMCELL Technologies) and passaged mechanically. Retinal organoid differentiation was carried out as previously reported with some modifications (Quinn et al., 2019; Zhong et al., 2014). Confluent hiPSCs were collected and incubated with (±)blebbistatin in mTeSR medium in micro-mold spheroids (Z764000-6EA, Merck) over night. Then, medium was transitioned to Neural Induction Medium 1 (NIM1) using mTeSR/NIM1 (3:1), then (1:1), and finally (0:1) over three days to form embryoid bodies (EBs). After 1 week, EBs were plated onto Matrigel-coated wells with daily NIM-1 medium change till DD15 and daily change of NIM-2 starting at DD16. Between DD20 and latest DD28, neuroepithelial structures were selected and flushed from the Matrigel plates using a P1000 pipet and kept in floating culture in agarose coated plates from this point onwards. After selecting the best-looking structures, all structures were flushed from the Matrigel plates and kept in floating culture to increase the yield of obtained organoids. Then, typically from DD40 until DD100, good retinal organoid structures were selected and placed individually in a 48 well plate. Brain and other non-retinal structures were removed as well between this time period. Daily medium change of NIM-2 is used till DD34, then typically three times a week Retinal Lamination Medium 1 (RLM-1) was used

from DD35 to DD63. Then, RLM-1 + 1μM retinoic acid until DD84, followed by RLM-2 + 0.5μM retinoic acid, and RLM-2 from DD120 was used for the rest of the culture.

Immunohistochemical analysis

Organoids were collected at DD180 or DD210 for immunohistochemical analysis. Organoids were fixed with 4% paraformaldehyde in PBS for 20 minutes at RT, briefly washed with PBS and subsequently cryo-protected with 15% and 30% sucrose in PBS until organoids sunk to the bottom of the well. Organoids were embedded in Tissue-Tek O.C.T. Compound (Sakura, Finetek), thereafter 8 μM cryosections were made with a Leica CM1900 cryostat (Leica Microsystems) and stored in the freezer.

For immunohistochemistry, the sections were blocked for 1h at RT in 10% normal goat serum, 0.4% Triton X-100, and 1% bovine serum albumin in PBS. Primary antibodies were incubated overnight at 4°C or for at least 3h at RT with 0.3% normal goat serum, 0.4% Triton X-100, 1% BSA and appropriate primary antibody concentration (Table S2). Then, slides were washed for two times 15 minutes in PBS and subsequently incubated for 1h at RT with fluorescent-labelled secondary antibody in 0.1% goat serum in PBS. Nuclei were counterstained with DAPI and mounted in Vectashield Hardset mounting medium (H1800, Vector laboratories, Burlingame, USA). Sections were imaged on a Leica TCS SP8 confocal microscope and images were processed with Leica Application suite X (v3.7.0.20979).

RNA isolation, cDNA synthesis, and qPCR analysis

RNA was isolated from DD210 retinal organoids of P116, P117, P128, ISO-P128, and ISO-02 P116 using TRIZOL reagent (Gibco Life Technologies) according to the manufacturer manual. The isolated RNA was dissolved in 20μl RNase-free water. 0.5μg of total RNA was reverse transcribed into first-strand cDNA using QuantiTect Reverse Transcription Kit (205311, QIAGEN) in a total reaction volume of 20μl. From all cDNA samples, a 1 in 20 dilution was made and used for qPCR analysis.

Two different exon-spanning primer pairs were designed at the 5' end of the *CRB1-B* gene giving rise to an amplicon of 70 to 120bp (FW1: TGTTTGGAGCCAGGACACAT, REV1: ACGTCTTCTTCGCAGTGGAT and FW2: GAGCCAGGACACATGGTTTTC, REV2: TTCCCAGGCAAGTTCTCACA). Real-time qPCR was based on the monitoring of SYBR Green I dye fluorescence on a CFX Connect Real-Time System (BioRad). The qPCR conditions were as follows: 5μl SYBR green PCR 2x master mix (4913914001, Merck), 0.2μl of 10μM FW and REV primers, and 5ul of the diluted cDNA. qPCR machine started with a melting step at 95°C for 10min, followed by 40 cycles of 95°C for 15 seconds and an annealing at 60°C for one minute. At the end of the PCR run, a dissociation curve was determined by ramping the temperature of the sample from 60 to 95°C while continuously collecting fluorescence data. MQ water controls were included for each primer pair to check for any significant levels of contaminants. The following two reference genes were used: glyceraldehyde 3-phosphate dehydrogenase (GAPDH) and elongation factor 1a (EF1A), previously described by (Pellissier et al., 2014).

qPCR was performed on both primer pairs with at least three individual DD210 retinal organoids of P116, P117, P128, ISO-P128, and ISO-02 P116 cDNA and human adult retina cDNA (Marathon-ready; Clontech). *CRB1-B* was detected in adult human retina cDNA but was below detection level in the patient-derived and isogenic control retinal organoids at DD210.

Single cell RNA sequencing

Retinal organoid dissociation

Retinal organoids were dissociated using an adapted protocol from the Papain Dissociation kit (Worthington, I-LK 03150). In short, single retinal organoids were selected and cut into small pieces to remove excess RPE or non-neural tissue as much as possible and placed on a 48 well plate with 500µl dissociation solution (20 units/ml papain and 0.005% DNase). These were incubated for 30 minutes on a shaker in the incubator, then the organoids were triturated using a 1mL pipettor to dissociate the tissue. The plate was placed back for 15-20 minutes on the shaker in the incubator, then again triturated, this was repeated until a single cell suspension was obtained. 500µl albumin ovomucoid protease inhibitor solution was added to the single cell suspension, centrifuged at 300x g for 5min. Supernatant was removed, pellet resuspend in PBS and filtered through a 40um cell strainer (Pluristrainer; SKU 43-10040-50).

Filtered single cell suspension was centrifuged at 300x g for 5min at 4˚C and resuspend in 100µl staining buffer (2%BSA/0.01%Tween, PBS) with 10µl Fc Blocking reagent (FcX, BioLegend) for 10 minutes on ice. 0.5µg of unique Cell Hashing antibodies were added and incubated for 20 minutes on ice. Stained cells were washed 3 times with 1mL staining buffer, spinned at 4 ˚C for 5 minutes at 350g. Stained single cell suspensions were counted for cell concentration and cell viability (TC20, Bio-rad). Typically, all single cell suspensions had a 70% or higher cell viability. Stained cells were pooled and re-counted until desired concentration for single cell sequencing (cell viability of at least 80%). Every sequencing round contained 14 hashed retinal organoid samples; the goal was to capture a total of approximately 30.000 cells per pool.

Droplet-based single-cell RNA sequencing

ScRNA-seq data was generated using the Chromium 10x 3'UTR-sequencing. Single cell suspensions were loaded onto the Chromium Single Cell system using the v3 chemistry. Subsequent steps were performed according to manufacturer's instructions.

Computational analysis of single cell data

Raw sequencing output were processed using the Cell Ranger (v6.0.1) pipeline (10X Genomics) with default settings and the pre-built human genome reference (GRCh38). Custom references of codon optimized AAV.h*CRB1*, codon optimized AAV.h*CRB2*, AAV.*GFP* and *CRB1-B* were added using the known FASTA sequence.

Filtered expression matrices were further processed with a Seurat (v4.1.0) based workflow in in R (v4.1.0) (Hao et al., 2021). In short, cells were demultiplexed based on their HTO enrichment using HTODemux function of Seurat, and singlets were selected for downstream analysis. Quality control

followed, keeping cells with nFeature_RNA > 800 and <6000, nCount_RNA <30000, and percent.mt <12. The raw counts were normalized with NormalizedData function (scale.factor = 30000). The top 2000 most variable genes were selected using FindVariableFeatures. Principal component analysis (PCA) was then performed using these 2000 genes. The first 15 PCs were used to calculate cell clusters and project the cells on a two-dimensional plot using Uniform Manifold Approximation and Projection (UMAP) algorithm. Top markers from FindAllMarkers function were analysed and compared to well-known cell type-specific markers to classify the clusters. For downstream analysis, data was subset per cluster, per patient derived retinal organoids, and/or per treatment. Then, differentially expressed genes were retrieved from the FindMarkers function. Genes with p_val_adj ≤ 0,05 were used for GO (Gene Ontology) term analysis, GO analysis was performed using g:Profiler (version e106_eg53_p16_65fcd97) with g:SCS multiple testing correction method applying significance threshold of 0.05 (Raudvere et al., 2019). Relevant terms and associated genes were included for visualization in this manuscript. For the violin plots comparing genes in *CRB1* patient-derived and isogenic control retinal organoids, the function stat_compare_means with a Wilcoxon t-test was used to determine statistically significant differences.

Number of cells used for downstream analysis per condition: (1) coming from one sequencing round, ISO-P116-*GFP*: *n*=993 cells from 4 organoids, P116-*GFP*: *n*=1497 cells from 4 organoids, P116-*CRB1-GFP*: *n*=1225 cells from 3 organoids, P116-*CRB2-GFP*: *n*=884 cells from 3 organoids, and (2) equally divided in three separate sequencing rounds, ISO-P128-*GFP: n*=5786 cells from 6 organoids, P128-*GFP*: *n*=4908 cells from 6 organoids, P128-*CRB1-GFP*: *n*=4386 cells from 5 organoids, P128-*CRB2-GFP*: *n*=3870 cells from 5 organoids, P117-*GFP*: *n*=3552 cells from 5 organoids, P117-*CRB1-GFP*: *n*=3108 cells from 5 organoids, P117-*CRB2-GFP*: *n*=2868 cells from 5 organoids.

Supplemental references

Hao, Y., Hao, S., Andersen-Nissen, E., Mauck, W.M., Zheng, S., Butler, A., Lee, M.J., Wilk, A.J., Darby, C., Zager, M., et al. (2021). Integrated analysis of multimodal single-cell data. Cell *184*, 3573-3587.e29. https://doi.org/10.1016/j.cell.2021.04.048.

Pellissier, L.P., Lundvig, D.M.S., Tanimoto, N., Klooster, J., Vos, R.M., Richard, F., Sothilingam, V., Garrido, M.G., Bivic, A. Le, Seeliger, M.W., et al. (2014). CRB2 acts as a modifying factor of CRB1-related retinal dystrophies in mice. Hum. Mol. Genet. *23*, 3759–3771. https://doi.org/10.1093/hmg/ddu089.

Quinn, P.M., Buck, T.M., Mulder, A.A., Ohonin, C., Alves, C.H., Vos, R.M., Bialecka, M., van Herwaarden, T., van Dijk, E.H.C., Talib, M., et al. (2019). Human iPSC-Derived Retinas Recapitulate the Fetal CRB1 CRB2 Complex Formation and Demonstrate that Photoreceptors and Müller Glia Are Targets of AAV5. Stem Cell Reports *12*, 906–919. https://doi.org/10.1016/j.stemcr.2019.03.002.

Raudvere, U., Kolberg, L., Kuzmin, I., Arak, T., Adler, P., Peterson, H., and Vilo, J. (2019). G:Profiler: A web server for functional enrichment analysis and conversions of gene lists (2019 update). Nucleic Acids Res. *47*, W191–W198. https://doi.org/10.1093/nar/gkz369.

Zhong, X., Gutierrez, C., Xue, T., Hampton, C., Vergara, M.N., Cao, L.H., Peters, A., Park, T.S., Zambidis, E.T., Meyer, J.S., et al. (2014). Generation of three-dimensional retinal tissue with functional photoreceptors from human iPSCs. Nat. Commun. *5*. https://doi.org/10.1038/ncomms5047.
